# Microdiversity Shapes the Seasonal Niche of Prokaryotic Plankton Inhabiting Surface Waters in a Coastal Upwelling System

**DOI:** 10.1111/1758-2229.70131

**Published:** 2025-07-21

**Authors:** Cessna‐Pamela Orta‐Ponce, Rodrigo Alba‐Salgueiro, Carlota Rodríguez, Joaquín Valencia‐Vila, Pilar Díaz‐Tapia, Antonio Bode, Mar Nieto‐Cid, Marta M. Varela

**Affiliations:** ^1^ Facultade de Ciencias Universidade da Coruña A Coruña Spain; ^2^ Centro Oceanográfico de A Coruña Instituto Español de Oceanografía IEO‐CSIC A Coruña Spain; ^3^ Departamento de Botánica, Facultade de Bioloxía Universidade de Santiago de Compostela Santiago de Compostela Spain

**Keywords:** diversity, DNA sequencing, ecological niche, flow cytometry, prokaryotes (Bacteria and Archaea), seasonality, upwelling

## Abstract

Seasonality of prokaryotic abundance, diversity and community composition was investigated over a 2‐year period in a coastal upwelling time‐series station. A marked seasonality was found for prokaryotic abundance, peaking during upwelling and upwelling‐to‐downwelling transition, and decreasing during downwelling. The latter included a deeper mixed layer and a homogeneous water column favouring higher abundance of archaea (i.e., Marine Group II, *Candiadatus nitrosopelagicus*), SAR406 clade and the group Bacteria_Others including > 400 rare taxa. Upwelling and transition conditions, characterised by enhanced vertical stratification and a marked hydrographic variability, included a community less diverse with core‐phylotypes proliferating, i.e., Flavobacteriaceae, Amylibacter and Planktomarina. Physical and biogeochemical variables collectively explained > 40% of the seasonal changes in prokaryotic assemblages. Additionally, fine‐tune bacterial features evidenced ‘closely related taxa’ within particular bacterial phylotypes such as SAR116 clade; certain Flavobacteria belonging to NS2b, NS4 or NS9; members of the family Cryomorphaceae and Marine Group II, displaying seasonal microdiversity patterns. Taken together, seasonal hydrographic forcing induces a shift in the upwelling‐driven microbiome providing new insights into the barely explored seasonal niche partitioning of surface prokaryotic communities in such highly productive upwelling systems. These results are of broad interest for understanding ecosystem functioning and forecast the impacts of current environmental change.

## Graphical abstract

Upwelling‐induced hydrological perturbation shapes seasonal transitions of prokaryotic plankton. Moreover, our results indicate seasonal keystone ecotypes and fine‐tuning of prokaryotic niche. This knowledge is of broad interest for understanding marine ecosystem functioning, and suggests the potential of environmental selection in the face of climate change scenarios.

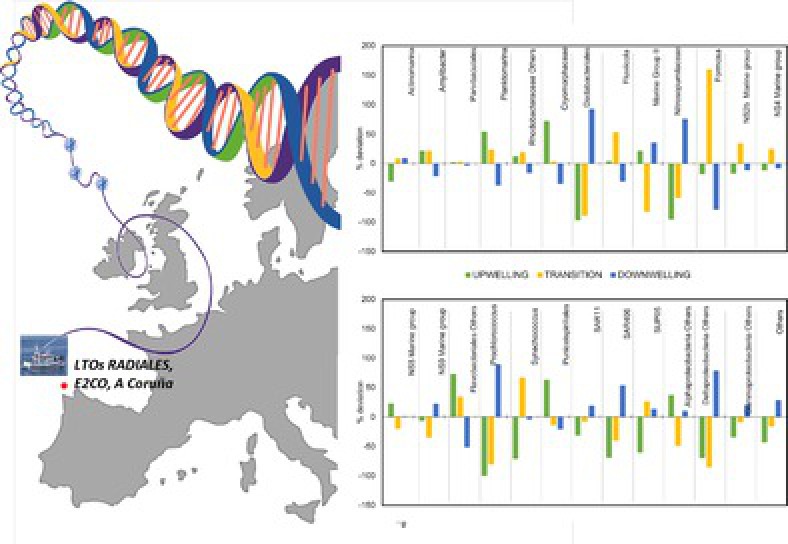

## 1 Introduction

Prokaryotes represent the most important biological group on earth in terms of phylogenetic and functional diversity. Also, they constitute a key component of the marine biome since they are key players in regulating the biogeochemical cycle of carbon (Gasol et al. 2009; Baltar et al. 2010; Herndl and Reinthaler 2013; Fuhrman et al. 2015; Evans et al. 2021; Muyzer and Cretoiu 2022), but also in the use and transformation of other elements like nitrogen or phosphorus that are essential for marine primary productivity (Acinas et al. 2022). The environmental conditions to thrive in coastal upwelling areas vary substantially in time because of the interactions between winds and the coastal topography. Nutrients reach the surface during spring and summer months (Bode and Varela 1998), whereas southerly winds are more frequent between autumn and winter, producing downwelling conditions and accumulating warm, less saline, and less dense shelf surface waters towards the coast (Bode et al. 2017; Montes et al. 2020). In addition, tidal forcing and freshwater inputs generate a considerable heterogeneity determining water exchanges and therefore fluxes of nutrients among the coastal zone (where upwelling occurs) and offshore waters (Decastro et al. 2000). Such conditions originate seasonal transitions, generating a system with high temporal environmental heterogeneity. Accordingly, microorganisms experience and respond to this temporal variability at many different biological and ecological levels. Still, microbiome shifts over time and their distribution patterns linked to environmental upwelling forcing as well as microbial taxa associations remain poorly explored despite their huge relevance to inform us on ecological niches of individual microbes in such heterogeneous upwelling systems (Fuhrman et al. 2006; Gilbert et al. 2012).

Ocean time‐series are beginning to discern the temporal variability and environmental drivers of marine microbiomes (Valdés et al. 2021). The combination of long‐term oceanographic time‐series (e.g., RADIALES in the NW of Spain, L2 Western English Channel, MOLA and SOLA in the NW Mediterranean region) together with the improvement and cost reduction in massive sequencing techniques has enabled the monitoring of prokaryotic communities through time, which is highly relevant to keep tracking these communities (of morphological simplicity) in order to forecast future behaviours in the face of a rapid climate change. In this sense, oceanographic time‐series studies have proven to be very useful to elucidate interactions between prokaryotic communities and environmental conditions (Lambert et al. 2021), displaying markedly repeatable seasonal patterns in the diversity of prokaryotes (e.g., Eiler et al. 2011; Gilbert et al. 2012; Cram et al. 2015; Fuhrman et al. 2015; Giner et al. 2019). Most importantly, some relatively recent studies linked to coastal oligotrophic time‐series stations have also shown seasonal variability within closely related taxa belonging to the same family/genera through oligotyping (e.g., Mena et al. 2020) or amplicon sequence variants (ASVs) (Auladell et al. 2021). In this regard, the ASV approach can now provide a reliable glimpse of closely related taxa showing seasonal variations in abundance and occurrence, distinguishing distinct ecotypes (adaptation of microbial phylotypes to different environmental conditions, e.g., Auladell et al. 2019). These ecotypes might likely play different ecological roles in the ecosystem and carry out different functions. Indeed, the realisation of this fine‐scale taxonomic differentiation has led to a debate on the ecological relevance of microbial taxa, and the need to define ecologically relevant units within higher microbial taxonomic levels (such as microorganism at subgenus level), since ecotype differentiation results in increased microdiversity and community stability. Thus, discriminating the seasonal ecotypes within particular prokaryotic taxa will resolve microbial communities' composition, diversity and in turn, metabolic functions in a more comprehensive manner, and will help to understand the response of the communities to environmental (such as the upwelling) perturbations. Unravelling the potential of environmental selection on ecotype divergence is particularly relevant in the light of climate change scenarios.

The Northwest Iberian upwelling system is a complex and dynamic environment where winds displace surface water off the coast, allowing nutrient‐rich deep water to reach the surface and drive remarkable productivity throughout all trophic levels supporting productive fisheries, aquaculture and coastal communities (Picado et al. 2023). Microorganisms inhabiting these ecosystems have the capability to function under extremely variable conditions (e.g., during specific perturbation events such as phytoplankton blooms in the northwestern Atlantic coast), and thus, play a disproportionately important role in the microbial‐mediated cycling of marine nutrients. Yet, microbial studies carried out in this dynamic upwelling site have limited temporal resolution, mainly taking place in a specific period (Montes et al. 2020) or lacking enough resolution (Hernando‐Morales et al. 2018). Here, we analysed a two‐year survey in the subsurface waters of A Coruña coast (NW Iberia) with the objective of (i) refining our understanding of the seasonal dynamics of prokaryotic communities at a fine ASV scale and further explore its link with oceanographic features under the influence of upwelling and (ii) investigating different ecological patterns within closely related specific taxa (i.e., ecotypes). We hypothesised that the heterogeneous oceanographic and biological features of coastal upwelling systems promote microdiversity and further support the temporal niche partitioning of certain prokaryotic phylotypes. Under the climate change scenario, a shift in environmental conditions has been predicted for this ecosystem, which is likely to affect regional productivity. Therefore, it is necessary to improve knowledge about the microbiome in this productive area, as it is the first step by which energy and material are transferred to higher trophic levels.

## 2 Methods

### 2.1 Sampling Strategy and Ancillary Physico‐Biogeochemical Measurements

Samples were obtained monthly at station E2CO (43° 25.3′ N, 8° 26.2′ W, 80 m depth, Figure S1) located in the continental shelf in front of the coast of A Coruña for a period of 2 years (from May 2016 to May 2018), within the framework of the time‐series project RADIALES (www.seriestemporales‐ieo.net). Prokaryotic and environmental samples used in all further analyses were collected from 0 m to ≈70 m depth. To study the seasonality of ancillary measurements (physical and biogeochemical variables) we used data from surface to ~75 m depth (Figure S2). Temperature and salinity profiles were measured using a Seabird‐25 conductivity‐temperature depth (CTD) sensor attached to a rosette. Aliquots for inorganic nutrients analysis (nitrate, nitrite, phosphate and silicate) were collected in polyethylene bottles and frozen at −20°C until measured in the home laboratory using a QuAAtro autoanalyser from SEAL Analytical. The protocols from SEAL analytics Q‐126‐12 and Q104‐09 were used for nitrate and nitrite, and Q‐125‐12 for phosphate concentration analysis (Coverly et al. 2012). Chlorophyll‐a, a, b, c1 + c2 and total concentrations (Chl‐a, Chl‐b, Chl‐c and TChl, respectively) was determined from acetonic extracts of plankton retained by GF/F filters and measured by the fluorometric method (Neveux and Panouse 1987). Primary production (PP) was measured by inoculating surface seawater with NaH^14^CO_3_, incubated under in situ water conditions for 2 h and filtered through GF/F filters following Bode et al. (2019). Particulate organic carbon (POC) and nitrogen (PON) was determined in 0.5–1 L of seawater filtered through Whatman GF/F filters, which was stored at −20°C until analysis in a Carlo Erba CHNSO 1108 analyser. The upwelling index (UI) is estimated, the volume of water transported by time unit and distance unit through an along‐shore transect from sea level atmospheric pressure fields (Gonzalez‐Nuevo et al. 2014). In this case, UI values were averaged over the 15 days prior to the DNA sampling dates from the Rías Altas time‐series (http://www.indicedeafloramiento.ieo.es/HAltas/). Precipitation data were obtained from the monthly accumulated rain of the Meteogalicia Coruña‐dique station (https://www.meteogalicia.gal/observacion/rede/redeIndex.action), averaging accumulated rain during 15 days prior to the DNA sampling.

### 2.2 Flow Cytometry

Prokaryotic abundance was determined monthly by flow cytometry following Montes et al. (2020). Samples were stained with Syto 13 (Life Technologies) in the dark for 10 min. Subsequently, prokaryotic cell counts were detected by their distinct signature in a plot of side scatter vs. green fluorescence using a FACSCalibur flow cytometer (Becton Dickinson). Finally, prokaryotes were displayed as total prokaryotic abundance (PA), percentage of high nucleic acid content prokaryotes (HNA_A) and *Synechococcus* abundance (Syn_A).

### 2.3 DNA Collection, Extraction, Amplification, Sequencing, and Bioinformatics Analysis

Seawater samples for DNA analyses were collected at 0 m depth by filtering 4–8 L through 0.22‐μm Sterivex filters (Millipore). Then, 1.8 mL of lysis buffer (40 Mm EDTA, 50 mMTRIS‐HCl, 0.75 M saccharose) was added to the cartridge filter and they were stored at −80°C until further analysis. The DNA extraction was performed following the phenol–chloroform extraction method described by Massana et al. (1997) with slight modifications. Cell lysis was performed by a 45‐min digestion with freshly made lysozyme (1 mg mL^−1^ final concentration) at 37°C, followed by a 60‐min proteinase K digestion (0.2 mg mL^−1^ final concentration) with sodium dodecyl sulfate (SDS) (10%) at 55°C. Then, DNA was purified twice with phenol: chloroform: IAA (25:24:1) and once with chloroform: IAA (24:1). The extracted DNA was concentrated using an Amicon Ultracel 100 k filter unit (Millipore). DNA concentration and purity were quantified according to the A260/A280 ratio using a Nanodrop spectrophotometer (Thermo Scientific, EEUU). Nucleic acid extracts were stored at −80°C until further analysis.

The V3 to V4 regions of the 16S rRNA gene were PCR amplified using the primer pairs 515F‐Y and 926R for Bacteria and Archaea (Parada et al. 2016). The 20‐μL PCR mixture contained 2 μL of the corresponding primer set (1 μL and 10 μM each), 2 μL 10 × PCR Buffer (Invitrogen), 1.2 μL MgCl2 (25 mM), 0.4 μL dNTP (10 mM), 1.25 U (0.025 U/μL) Taq polymerase (Platinum, Invitrogen) and 1 μL of DNA template (~20 ng) and completed with sterilised ultrapure water. PCR amplification was performed by using a BIO‐RAD T100 Thermal Cycler. Cycling conditions for amplification of DNA were 94°C, 3 min; (25 cycles of 94°C, 60 s; 50°C, 60 s; 72°C, 105 s and 72°C, 10 min) (Parada et al. 2016). PCR products were checked for quality control on a 1% (w/v) agarose gel electrophoresis, cleaned and purified with 5‐Prime ArchivePure purification kit (Fisher Scientific), and kept at −20°C until further analysis.

Amplicons were analysed in an Illumina Miseq platform using 2 × 250 bp paired‐end runs. From raw sequence data, primers and spurious sequences were trimmed using cutadapt. The ASVs were differentiated using the dada2 (Callahan et al. 2017) pipeline implemented in QUIIME2 (http//qiime2.org). The approach is threshold‐free, inferring exact variants up to one nucleotide of difference using the Q scores in a probability model. This pipeline was implemented through the high‐performance supercomputing resources belonging to the Centro Tecnolóxico de Supercomputación de Galicia (CESGA). Sequences were aligned against SILVA 132 16S rRNA database (Quast et al. 2013) as reference. Finally, singletons (ASVs found only once in the final ASV table) were excluded, as they have been shown to be likely the result of PCR or sequencing errors (Huse et al. 2010). Before rarefaction, chloroplast and mitochondria sequences were eliminated from the database, presenting a total number of reads of 939,873 ranging from 25,370 to 61,602 reads in each sample. The dataset was thus rarefied to the lowest number of reads per sample (25,370 reads) to enable diversity comparisons among samples. Then, ASVs were grouped into core‐phylotypes following the criteria of presenting a relative abundance ≥ 0.1%. For the analysis of closely related taxa at ASV level, a total of 25 core‐phylotypes were established based on the following criteria: appearing in at least 50% of all samples and presenting a relative abundance ≥ 0.25% with respect to the whole dataset. ASV richness and diversity metrics were determined with the function estimate R (vegan package, Oksanen 2018) in R (R Core Team 2018).

### 2.4 Statistical Analyses

Constrained ordination techniques were utilised to identify patterns of variation in prokaryotic community structure and correlations between prokaryotic community structure and environmental descriptors. All analyses of environmental and biological data were performed using the software package PRIMER v7 and PERMANOVA+ (Clarke and Gorley 2015) unless otherwise specified. First, environmental variables were tested for multicollinearity by analysing Draftsman plots and variables to be used in further analyses were reduced to: NO_3_—NO_2_, SiO_2_, PO_4_, temperature, salinity, PP, precipitation, POC, PON, and TChl. Subsequently, the environmental variables were log transformed and normalised prior to analysis, then the Euclidean distance similarity matrix was calculated. For the biological data, square root transformation was performed and then Bray–Curtis similarity was calculated. Distance‐based linear modelling (DistLM) was conducted to determine the environmental variables explaining the observed similarity among the prokaryotic assemblages. This procedure utilises a permutational approach, whereby the similarity between samples was calculated using the Bray–Curtis similarity measure. The Akaike Information Criterion (AIC) was used to select the predictor variables. The contribution of each environmental variable was assessed, firstly using ‘marginal test’ to assess the statistical significance and percentage contribution of each variable taken alone, and secondly, a ‘sequential test’ was employed to evaluate the cumulative effect of the environmental variables explaining assemblages' similarity. Distance‐based redundancy analysis (dbRDA) was generated to visually display the direction and magnitude of the relationship between environmental variables and the associated community of each environmental feature (upwelling, upwelling‐to‐downwelling transition and downwelling). Additionally, PERMANOVA tests (PERMANOVA + add‐on, Anderson et al. 2008) were conducted to assess the similarity between prokaryotic assemblages of the three environmental features (upwelling, upwelling‐to‐downwelling transition and downwelling).

The Shannon diversity (H′) and Chao richness estimator (S.Chao1) were calculated in R (R Core Team 2018) with the vegan package (Oksanen 2018). To assess the correlation of environmental features and taxa at both core‐phylotype and ASV levels, the normality of the variables was tested with the Shapiro–Wilk test (Shapiro and Wilk 1965) in R (R Core Team 2018). Then, Pearson correlation tests (Pearson 1920), performed in XLSTAT (Addinsoft 2020), were used to determine the bivariate correlation between core‐phylotypes and/or ASVs and the environmental variables. The correlation matrix was afterward represented in heatmaps using Euclidean distance hierarchical clustering in XLSTAT (Addinsoft 2020).

## 3 Results

### 3.1 Physical and Biogeochemical Oceanographic Conditions

**FIGURE 1:**
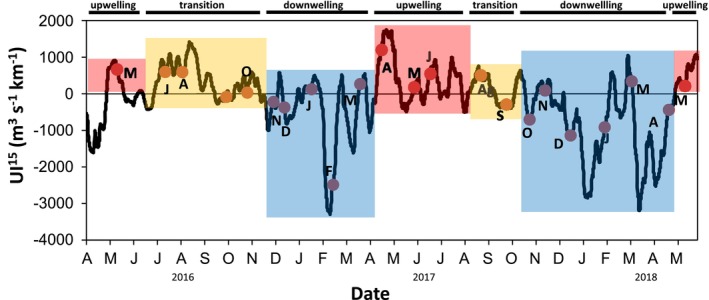
Fortnight averages of the upwelling index (m^3^ s^−1^ km^−1^) at one 1 × 1° grid centred at 44° N, 9° W during the sampling period covering from May 2016 to May 2018. Original data provided by IEO (http://www.indicedeafloramiento.ieo.es/HAltas/). Dots in the line correspond with sampling dates. Colour shadow areas and letters on the top indicate the upwelling, downwelling and transition periods. Dashed line indicates the break among years 2016, 2017 and 2018.

The distribution of the monthly upwelling index (UI) during the sampling period was characterised by upwelling pulses of variable intensity (as inferred from positive values) from May to October 2016, April to September 2017, and May 2018 (Figure 1 and Table S1). Downwelling conditions (as inferred from negative values) predominated from November to March 2017 and from October to April 2018 (Figure 1 and Table S1). The thermohaline variability throughout the sampling period was essentially driven by the typical seasonal thermal cycle, with a vertical gradient in temperature at the subsurface from June to October and a thermohaline mixing from November to April (Figure S2). From March to August, strong upwelling pulses introduced deep and cold waters near the surface, bringing high concentrations of inorganic nutrients, allowing the formation of phytoplankton blooms (Figure S2). The highest concentrations of Chl‐a are found from March to November, coinciding with peaks of PON and the increase of nutrients (e.g., NO_3_) (Figure S2 and Table S1). The relatively low salinity (< 34.6) found in surface waters during February and March strongly supports the influence of freshwater runoff in A Coruña, as inferred from accumulated precipitation data for both years (Figure S3 and Table S1). Based on this hydrographic variability, stations were classified into upwelling, downwelling, and upwelling‐to‐transition from upwelling to downwelling periods. To facilitate the comparison among hydrographic conditions, we provide mean and standard deviation (Table 1) for upwelling, upwelling‐to‐transition and downwelling periods.

**TABLE 1:** Average environmental characterisation during the upwelling, transition and downwelling periods from May 2016 to May 2018. Variables are seawater temperature, salinity, density, nutrients (NO_3_, NO_2_, PO_4_ and SiO_2_), chlorophyll‐a, b, c, and total concentrations (Chl‐a +Chl‐b +Chl‐c, TChl), primary production (PP), particulate organic carbon (POC), particulate organic nitrogen (PON), and particulate organic carbon and particulate organic nitrogen ratio (C:N ratio), upwelling index (UI) and precipitation.

	Upwelling (*n* = 5)	Transition (*n* = 6)	Downwelling (*n* = 11)
Temperature (°C)	14.87 ± 0.73	15.75 ± 0.40	13.83 ± 0.27
Salinity	35.48 ± 0.08	35.50 ± 0.03	35.47 ± 0.10
Density (kg m^−3^)	26.36 ± 0.14	26.19 ± 0.09	26.58 ± 0.09
NO3 (mmol kg^−1^)	0.78 ± 0.73	1.72 ± 0.60	4.35 ± 0.65
NO2 (mmol kg^−1^)	0.06 ± 0.01	0.11 ± 0.04	0.33 ± 0.06
PO4 (mmol kg^−1^)	0.10 ± 0.03	0.19 ± 0.05	0.22 ± 0.04
SiO2 (mmol kg^−1^)	0.61 ± 0.14	1.47 ± 0.38	2.56 ± 0.34
Chl‐a (mg m^−3^)	3.02 ± 1.11	1.81 ± 0.44	0.83 ± 0.19
Chl‐b (mg m^−3^)	0.24 ± 0.06	0.24 ± 0.06	0.17 ± 0.02
Chl‐c (mg m^−3^)	0.52 ± 0.20	0.27 ± 0.07	0.13 ± 0.04
TChl (mg m^−3^)	3.78 ± 1.35	2.32 ± 0.52	1.14 ± 0.25
PP. (mgC m^−3^ h^−1^)	14.28 ± 9.19	10.17 ± 4.94	8.06 ± 2.84
POC (mmol m^−3^)	21.30 ± 6.32	16.58 ± 4.88	7.57 ± 0.90
PON (mmol m^−3^)	2.57 ± 0.62	2.19 ± 0.45	0.98 ± 0.10
C:N ratio (molar)	8.7 ± 1.43	7.27 ± 0.52	7.73 ± 0.34
UI (m^3^ s^−1^ km^−1^)	559.42 ± 184.61	224.21 ± 158.30	−496.75 ± 246.68
Precipitation (L m^−2^)	12.08 ± 4.88	14.72 ± 6.19	31.38 ± 8.03

### 3.2 Seasonal Patterns of Microbial Abundance

**FIGURE 2:**
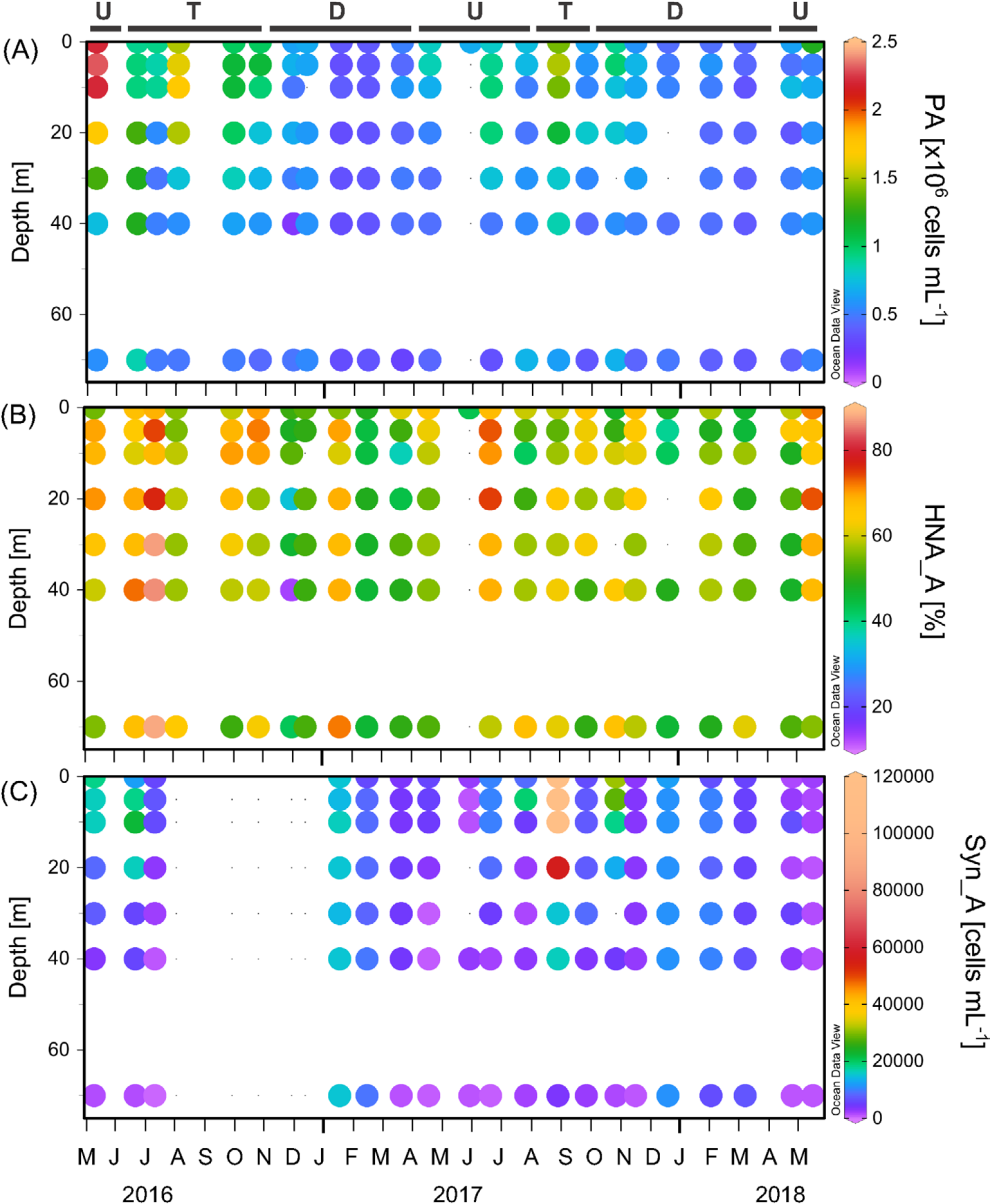
Prokaryotic abundance, PA (A), high nucleic acid content prokaryotes abundance in percentage, HNA_A (B) and *Synechococcus* sp. abundance (C) from subsurface water samples at station E2CO. Letters above the bars indicate the prevailing hydrographic periods: U = upwelling, T = transition and D = downwelling.

Prokaryotic abundance was generally low in autumn‐winter, ranging from 2.20 × 10^4^ to 7.90 × 10^5^ cells mL^−1^ in autumn and from 1.60 × 10^5^ to 7.70 × 10^5^ cells mL^−1^ in winter, whereas the highest values were measured in spring and summer, reaching up to 2.3 × 10^6^ cells mL^−1^ (Figure 2). Total prokaryotic abundance and high nucleic acid content cell abundance peaked during the upwelling pulses and decreased during the upwelling‐to‐downwelling transition, showing the lowest values during the downwelling periods. The abundance of high nucleic acid content cells clearly dominated during the spring–summer period (Figure 2). *Synechococcus* displayed high abundances during hydrographic conditions associated with the upwelling relaxation (Figure 2).

### 3.3 Seasonal Variations of Prokaryotic Diversity and Core‐Community Composition

Sequencing of 22 samples from station E2CO in A Coruña yielded 939,873 reads after quality curation of sequences and after removing chloroplast and mitochondrial sequences. Then, sequences were classified into 1523 amplicon sequence variants (ASVs) across the 2‐years study. To account for different sequencing depths, reads were normalised to the minimum number of reads per sample (25,370 reads) resulting in a total of 558,140 reads and 1506 different ASVs are ranging from 172 to 491 ASVs per sample.

**FIGURE 3:**
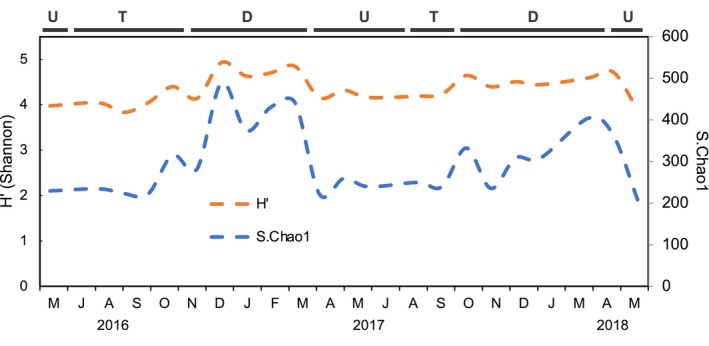
Seasonal variability of the Shannon diversity index (H′) and estimated ASV richness (S.Chao1) of prokaryotes for the period of study. Letters above the bars indicate the prevailing hydrographic periods: U = upwelling, T = transition, and D = downwelling.

Temporal variations in species richness estimator S.Chao1 and Shannon diversity index (H′) were determined by calculating the observed number of ASVs. Similar seasonality trends in richness and diversity were observed by both indices during the survey, with minimum values during the upwelling and upwelling‐to‐downwelling transition (May to Sep 2016 and 2017) and maxima (Dec‐16, Feb‐17, Mar‐18 months) during downwelling (Figure 3). On average, downwelling periods showed higher richness and diversity (364.05 ± 76.67 and 4.63 ± 0.22, respectively) than upwelling pulses (234.05 ± 19.89 and 4.13 ± 0.17) and upwelling‐to‐downwelling transition (250.14 ± 35.16 and 4.15 ± 0.20) (Figure S4). Both species richness and diversity were significantly different among the upwelling and the downwelling (*t*‐test, two‐tailed *p* = 0.0025; t‐test, two‐tailed *p* = 0.0006, respectively) and among the upwelling‐to‐downwelling transition and the downwelling (t‐test, two‐tailed *p* = 0.0039; t‐test, two‐tailed *p* = 0.0005, respectively) periods, while the upwelling and the upwelling‐to‐downwelling transition were not significantly different from each other (*t*‐test, two‐tailed *p* > 0.05).

**FIGURE 4:**
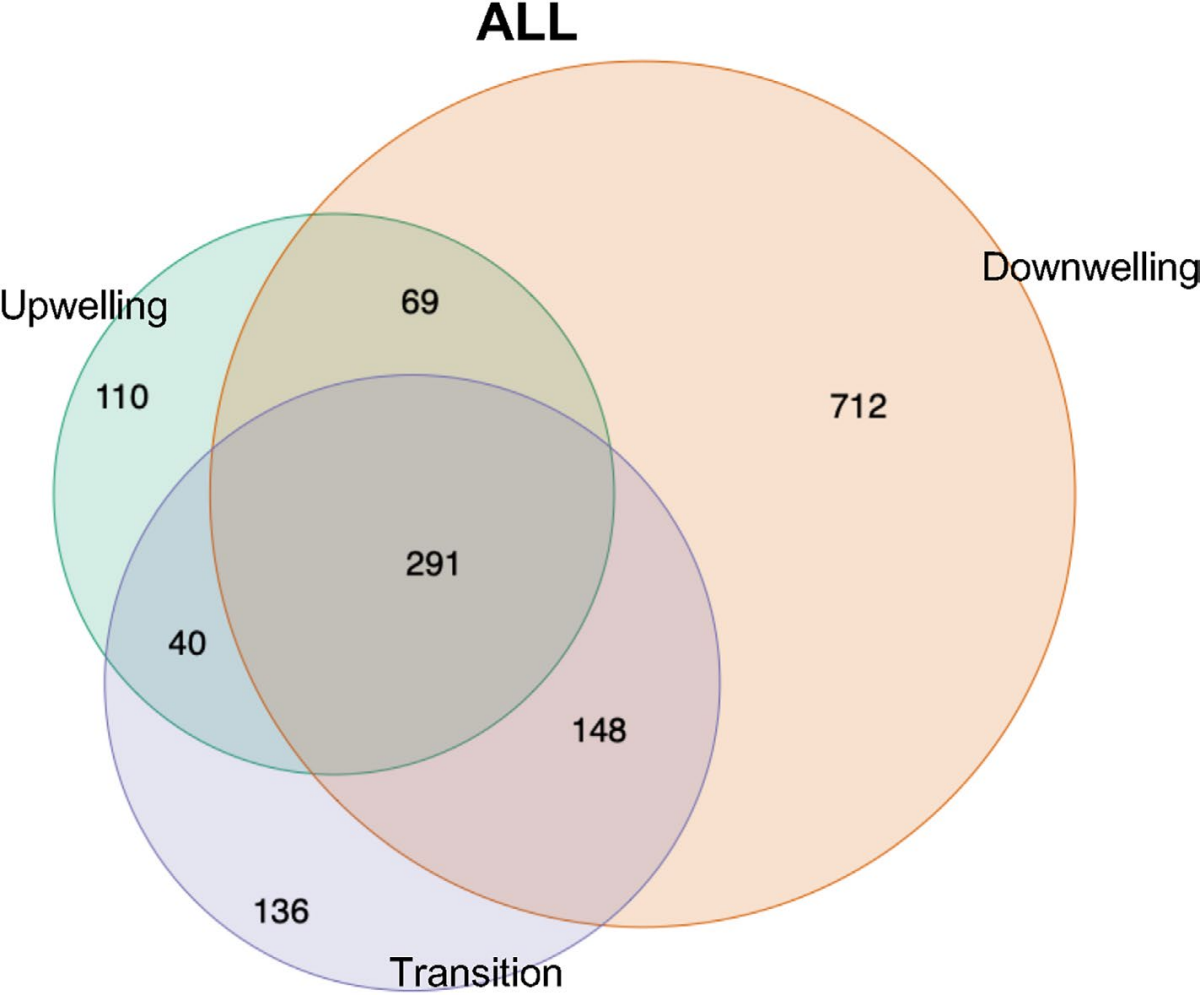
Venn diagram of all the prokaryotic communities between environmental events: upwelling, transition and downwelling. Samples were normalised to the minimum number of reads found in one of the samples (25,370 reads). Samples of each environmental event were averaged for each ASV. The Venn diagram illustrates the overlap of prokaryotic ASVs corresponding to upwelling, transition and downwelling events. The numbers in the circles indicate the number of unique ASVs in each event and the shared number of ASVs in the overlapping environmental events.

Venn diagram of the whole prokaryotic community (Figure 4) showed that periods of downwelling had the largest number of unique ASVs (712/1506 ASVs) compared to upwelling and upwelling‐to‐downwelling transition (110/1506 and 136/1506 ASVs), respectively. A total of 69 ASVs were shared among upwelling and downwelling, 148 ASVs among downwelling and upwelling‐to‐downwelling transition, and 40 ASVs among upwelling and upwelling‐to‐downwelling transition (Figure 4). Analysis of this microbiome time‐series data showed that 23% (352 ASVs) coexists throughout the 2‐year period of study, and these persistent ASVs contributed to a 90% of all reads. Taxonomic classification of these reads revealed that they mainly contributed to core‐phylogenetic groups (abundant ASVs) described below (Figures 4, [Link], [Link]).

The dbRDA analysis identified three separate groups/clusters based on the community similarity: downwelling samples cluster separately from those collected during the upwelling pulses and the upwelling‐to‐downwelling transition period (Figure S7). This separation into three specific communities was further supported by the PERMANOVA test: upwelling versus downwelling (*p* = 0.001), upwelling‐to‐downwelling transition versus downwelling (*p* = 0.003) and upwelling‐to‐downwelling transition versus upwelling (*p* = 0.006).

Redundancy analysis (dbRDA) indicated that the samples were divided into three different groups according to several significant environmental variables. Inorganic nutrients (NO_3_—NO_2_, SiO_2_, PO_4_), temperature, salinity, PP, precipitation, particulate organic matter (POC and PON) and TChl explain ~42% of the total variation of prokaryotic assemblages in the first two axes (*R* ^2^ = 0.3967, Figure S7 and Table S2). Overall, temperature represented the primary factor driving prokaryotic assemblages, showing a clear separation among upwelling/upwelling‐to‐downwelling transition and downwelling assemblages. Further on, UI separated upwelling from transition assemblages (Table S2). Along axis 1, samples in the downwelling cluster were on the left part of the ordination map, and associated with inorganic nutrients. Along axis 2, most of the samples in the upwelling‐to‐downwelling transition cluster (with the only exception of Oct 16) were on the lower part of the ordination, associated with PP, salinity and PON, while the upwelling cluster samples were on the upper part, associated with precipitation, TChl and POC.

Inorganic nutrients showed a negative relationship with both axis 1 and axis 2, influencing the microbiomes of downwelling conditions. Precipitation, TChla, and POC displayed a positive relationship with axis 1 and 2, which were shaping the prokaryotic community assembly of upwelling pulses. PON showed a positive relationship with axis 1 while negative with axis 2, and mainly influenced the assemblages of upwelling‐to‐downwelling transition period. UI was positively related with axis 1 and neither positively nor negatively related to axis 2, consequently influencing both upwelling and upwelling‐to‐downwelling transition assemblages.

**FIGURE 5:**
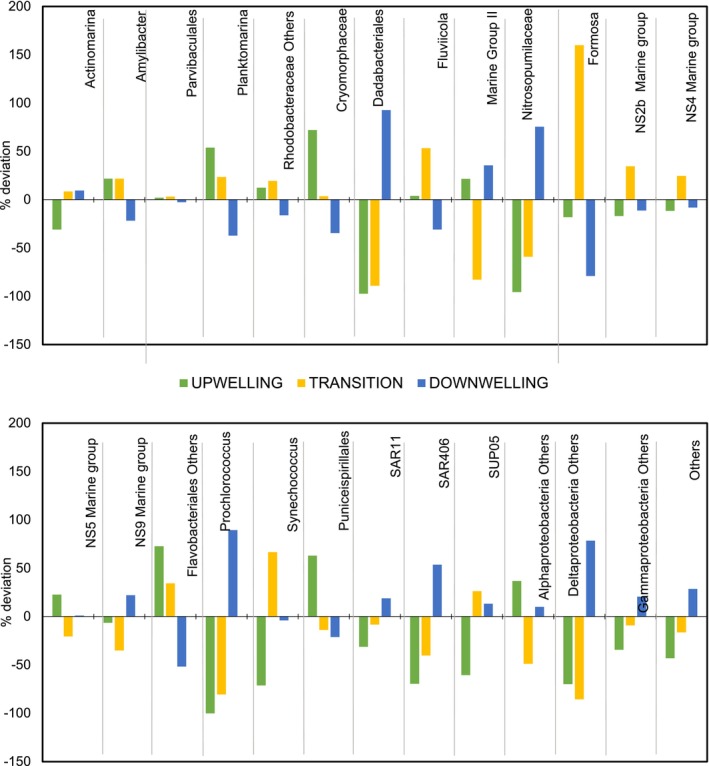
Deviation (in %) from the mean relative abundance of core phylotypes in upwelling, transition and downwelling events. The relative abundance of each event was calculated as the average relative abundance of the upwelling, transition and downwelling months, respectively.

The taxonomic classification of reads showed that the prokaryotic community composition was dominated by bacteria over archaeal sequences, and that bacterial contribution was thoroughly more consistent throughout the year (mean ± SD = 86.56% ± 13% per month) than archaeal contribution (13.43% ± 83.33%). The archaeal community was composed of family Nitrosopumilaceae (Thaumarchaeota) and Marine Group II (Euryarchaeota), mostly (99.46% of all archaeal reads). The bacterial community was composed of well‐known seawater groups such as *Actinomarina*, *Amylibacter*, Flavobacteriales, SAR11, SAR406, SUP05 and the cyanobacteria *Synechococcus* and *Prochlorococcus*. Sequences classified as Bacteroidia showed the highest relative abundance and comprised 34.04% of all sequences, followed by the Alphaproteobacteria (30.74%). The relative contribution within this group showed a substantial variation among different temporal communities. Flavobacteriales_Others mainly dominated during the upwelling pulses (3.50% of all reads and 39.23% of all Flavobacteriales_Others reads) and upwelling‐to‐transition periods (3.26% and 36.63%, respectively) together with the Alphaproteobacteria *Amylibacter* accounting for 2.31% of all reads and 27.68% of all *Amylibacter* reads in upwelling, and 2.77% and 33.19% in upwelling‐to‐downwelling transition, respectively. The archaea Marine Group II was also increased during upwelling pulses (Figure 5), as well as during the downwelling period (accounting up to 5.42% of the whole reads and 67.71% of all Marine Group II reads). Other taxa, such as Dadabacteriales, the archaea Nitrosopumilaceae, cyanobacteria *Prochlorococcus*, SAR406 and Deltaproteobacteria_Others were peaking remarkably during the downwelling period, whereas in upwelling and upwelling‐to‐downwelling transition periods their abundances decreased (Figure 5). In contrast, *Amylibacter*, *Planktomarina*, Cryomorphaceae, *Fluviicola* and Flavobacteriales_Others decreased markedly during downwelling while in upwelling and upwelling‐to‐downwelling transition period the abundance of these groups tends to increase (Figure 5). Additionally, core‐phylotypes showing equally large seasonal abundances do not display wide differences seasonally in their abundance among hydrographic features (upwelling, upwelling‐to‐downwelling transition and downwelling). Additionally, Parvibaculales and Flavobacteriales_*NS4 and _NS5* were persistent core‐phylotypes, showing no significant differences throughout the year (Figures 5, [Link], [Link]).

### 3.4 Seasonal Variability of Prokaryotic Community Composition at ASV‐Level

**FIGURE 6:**
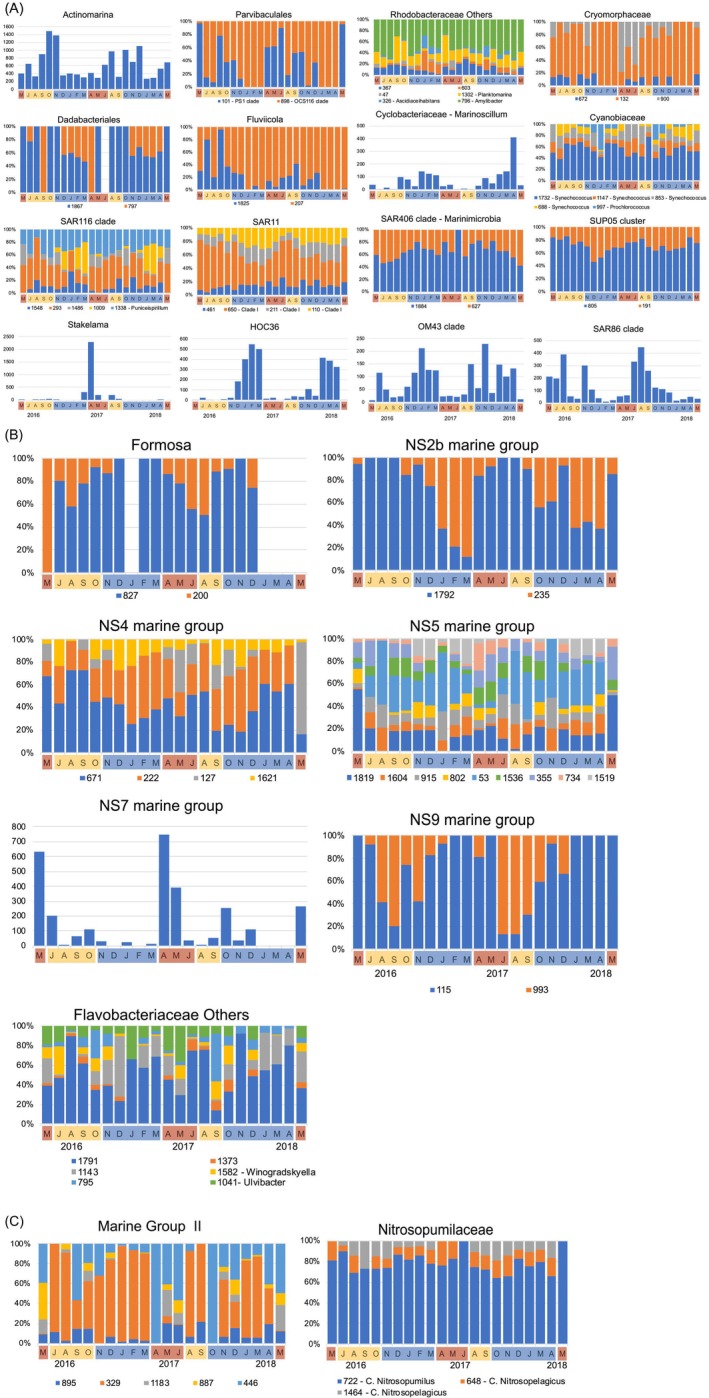
(A) Seasonality of *Phylum* Bacteria closely‐related taxa in the period of study. Graphs with a single ASV display the number of reads in each sample while graphs with two or more ASVs display the relative abundance of each ASV in each sample. Labels denote the ASV ID number. Colour shadow boxes and letters on the bottom indicate the upwelling (red), downwelling (blue) and transition (yellow) periods. (B) Seasonality of *Order* Flavobacteriales in the period of study. Graphs with a single ASV display the number of reads in each sample while graphs with two or more ASVs display the relative abundance of each ASV in each sample. Labels denote the ASV ID number. Colour shadow boxes and letters on the bottom indicate the upwelling (red), downwelling (blue) and transition (yellow) periods. (C) Seasonality of *Phylum* Archaea in the period of study. Graphs with a single ASV display the number of reads in each sample while graphs with two or more ASVs display the relative abundance of each ASV in each sample. Labels denote the ASV ID number. Colour shadow boxes and letters on the bottom indicate the upwelling (red), downwelling (blue) and transition (yellow) periods.

With the aim of exploring differences among ‘closely related phylotypes’, core‐phylotypes were broken down to ASV level to visualise its fine‐tuning occurrence and relative abundance during the 2 years of the study (Figure 6A–C). Core‐phylotypes displaying a more heterogeneous pattern at subgenus level returned the highest number of closely related ASVs, such as Flavobacteriales_*NS5*, Rhodobacteraceae and Flavobacteriaceae_Others including 9, 6 and 6 closely related ASVs, respectively. Furthermore, the occurrence and relative abundance over the course of the different seasons varied depending on the core‐taxa. For instance, ASV127, ASV900 and ASV293 belonging to Flavobacteriales_*NS4*, Cryomorphaceae and SAR116 clade, respectively, were associated to the hydrographic signature of upwelling pulse shifting, whereas their closely related relatives ASV222 belonging to Flavobacteriales_*NS4*, ASV132 belonging to Cryomorphaceae, and ASV1009 belonging to SAR116 clade, may prefer to thrive during hydrographic periods of water column mixing occurring during downwelling period (Figure 6A,B). Some other examples of seasonal heterogeneity include the Flavobacteriaceae_*NS2b* dominant ASV1792 (Figure 6B) and Dadabacteriales ASV1867 decreasing during downwelling period, whereas their relative abundance was highest during upwelling and upwelling‐to‐downwelling transition period. Similarly, ASV898 belonging to Parvibaculales OCS116 clade showed high relative abundance during the upwelling‐to‐downwelling transitional period. By contrast, the opposite trend was found for ASV827 belonging to *Formosa* and ASV115 belonging to Flavobacteriales NS9 occurring in high relative abundance during downwelling compared to upwelling and upwelling‐to‐downwelling transition period. In the same way, ASV1884 belonging to SAR406 and ASV191 belonging to SUP05 *cluster* tends to increase in relative abundance during downwelling periods, whereas ASV627 belonging to SAR406 and ASV805 belonging to *SUP05 cluster* show highest abundances during upwelling pulses (Figure 6A). Also, specific members of the other major prokaryotic group, Archaea MGII, such as ASV446, tends to increase during upwelling pulses, whereas ASV329 is dominating Marine Group II community during dowelling conditions (Figure 6C). Besides, core‐phylotypes described only by a single ASV showed also distinctive abundance seasonal patterns during the 2 years of study, such as the increased abundance during the downwelling period showed by HOC36 and *OM43 clade* and the increased abundance of SAR86 clade during upwelling and upwelling‐to‐downwelling transition period (Figure 6A).

### 3.5 Seasonal Prokaryotic Dynamics Linked to Environmental Factors

**FIGURE 7:**
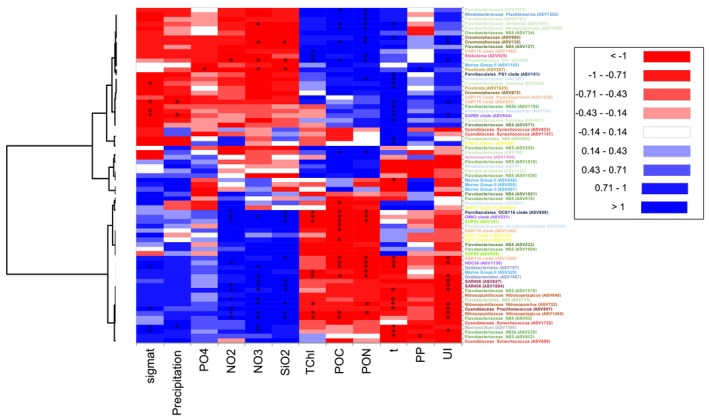
Heatmap shows correlations among environmental and biological variables, and individual ASVs. The colour gradient indicates positive correlations in blue tones and negative correlations in red tones, while white indicates no correlation. Significant correlations were highlighted with asterisks. Clusters show the aggregation of prokaryotic groups based on their similarity using Euclidian distance.

As some of the observed phylogenetic groups could just reflect either shared or distinctive environmental requirements, we explored the separation among co‐occurrence ASVs at both core‐phylotype (Figure S8) and ASV level (Figure 7) by analysing their relationship with the environmental factors. Our results displayed two main ecological interplays between the prokaryotic microbiome and biotic and abiotic factors. Downwelling prokaryotic assembly correlated with inorganic nutrients, seawater density and precipitation, while upwelling and upwelling‐to‐downwelling transition assembly correlated with organic material, upwelling index and temperature (Figures 7 and S8). Overall, core‐phylotypes such as Dadabacteriales and Nitrosopumilaceae showed negative correlations with organic material (POC and PON), temperature and UI, and positive correlations with precipitation and NO_3_. By contrast, *Formosa* and Cryomorphaceae correlated negatively with precipitation, NO_3_, PO_4_ and SiO_2_, while they showed strong positive correlations with UI and PON (Figure S8).

Interestingly, these abovementioned significant correlations among core‐phylotypes and environmental variables differed at ASV level (see Figure 7), showing a certain intra‐variability within each core‐phylotype linked to environmental variables. For instance, ASV53, ASV802 and ASV1519 (*NS5*) belonging to Flavobacteriaceae showed a negative relationship with temperature, UI, chlorophyll and particulate material, while other Flavobacteriaceae ASVs displayed opposite patterns (e.g., ASV734 and ASV355). Moreover, four of the five ASVs identified as Marine group II (ASV895, ASV446, ASV887 and ASV1183) correlated negatively with PO_4_, while ASV329 showed a strong positive correlation with PO_4_. Additionally, ASV446 and ASV329 belonging to Marine Group II showed a negative relationship with temperature and particulate material, respectively, while ASV1183 showed the opposite pattern. Other downwelling taxa belonging to the SAR116 clade were also a heterogeneous group with several and different correlations with the biotic and abiotic factors depending on the ecotype. For instance, ASV1009 and ASV1548 belonging to the SAR116 clade present a negative correlation to chlorophyll and particulate matter, as well as to temperature and UI, respectively. By contrast, ASV293, ASV1486 and ASV1338 (*Puniceispirillum*) belonging to SAR116 show a positive relationship with temperature and UI.

## 4 Discussion

### 4.1 Prokaryotes Are Biomarkers of Seasonal Upwelling Disturbances

Prokaryotic communities are diverse and dynamic, and knowledge of the factors controlling the success of individual taxa is important for understanding microbial influence on ecosystems and biodiversity. Although some prokaryotic community variation in this area was predictable using coarse phylogenetic characterisation (Guerrero‐Feijóo et al. 2017; Montes et al. 2020), disturbances such as upwelling may require more finely resolved prokaryotic community characterisation. Here we explored whether ecological interpretation of in situ dynamics of prokaryotic assemblages influenced by upwelling events would benefit from high‐resolution sequencing analytical methods (Callahan et al. 2017). Indeed, our analysis provides novel information about niche divergence of closely related taxa and their connectivity patterns to upwelling‐related environmental disturbances including shared or distinctive microbial taxa, suggesting temporal niche partitioning and fine‐grained of the realised niche of closely related taxa. A clear example is found in the *Flavobacterium* genus belonging to Bacteroidetes, showing in the present investigation that closely related ASVs could represent distinct taxa for some bacterial groups occupying temporally different niches, while in previous studies, we could only evidence Bacteroidetes and the family Flavobacteriaceae associated with changes in the concentration of Chl‐a dominating during the phytoplankton bloom (Valdés et al. 2021; Montes et al. 2020) with no distinctions among subspecies and/or species. Thus, we underline the importance of deciphering microbial communities, beyond common phylogenetically assigned core taxa, because of the complex interdependence among different physicochemical features and the multitudinous ecological processes mediated by different marine microbial components. In the following sections, we will discuss some of these findings to understand how upwelling reshapes microbial abundance and community composition.

### 4.2 Prokaryotic Abundance Is Boosted by the Upwelling

During upwelling, nutrient‐rich waters are physically transported to the surface, which initiates a cascade of energy transfer processes from microorganisms to other organisms in the upper food web through phytoplankton and zooplankton (Bode et al. 2009). In accordance with previous studies, upwelling has been found to have significant influences on the abundance of prokaryotes (Valencia et al. 2003). A coupling between available nutrients and temperature could be responsible for explaining the increase of its abundance during upwelling conditions in comparison with the downwelling season (Figueiras et al. 2006; Liu, Xie, et al. 2022; James et al. 2022). Indeed, temperature was identified as the most important factor shaping the microbial prokaryotic community, as core‐prokaryotic groups and closely related ASVs within core‐taxa were found to have close correlations with temperature (Figures 7 and S8). Additionally, the rise of NO_3_ from the bottom to the surface favoured phytoplankton blooms at the beginning of each upwelling pulse (as phytoplankton utilises nitrate and in turn the nitrate pool diminishes). Soon after that, both PON and the concentration of Chl‐a increase (Savoye et al. 2003), functioning as a bioreactor of organic matter. This pileup of organic matter was shown by high levels of Chl‐a during upwelling and to a lesser extent during the upwelling‐to‐downwelling transition period (see Figure S2) that might likely fuel prokaryotes. Furthermore, at the beginning of spring in both 2017 and 2018, accumulated rain peaks may add important inputs of organic material and nutrients (Figure S3) to the surface that also facilitate the increase in abundance of total prokaryotes. Although the abundance of Cyanobacteria decreased in the early stages of upwelling, it dominated the microbial community during the upwelling‐to‐downwelling transition period likely suggesting they contribute to carbon uptake and subsequent export to the adjacent ocean by upwelling filaments (Álvarez‐Salgado et al. 2007). Overall, we speculate that prokaryotes enact a rapid and direct response in terms of abundance to engage with a heterogeneous upwelling system.

### 4.3 Upwelling‐Related Disturbances Shape the Seasonal Dynamics of Core‐Prokaryotic Assemblages

Prokaryotic diversity and community composition assessed throughout 2 years of sampling exhibited considerable seasonal changes. Upwelling events usually bring abundant nutrients with low water temperatures, which could be a selection pressure (cold stress) that eventually affects microbial diversity (Montes et al. 2020). In this way, the analysis of alpha diversity revealed a marked seasonality diversity trend, showing maximal Shannon diversity and richness during downwelling, also supported by the highest number of unique ASVs (712 ASVs) displayed during this period. During the downwelling season, the change of the winds to poleward direction reverses the circulation of currents, pushing surface coastal waters down to the bottom (Lorente et al. 2020) allowing oxygen to reach deeper waters. As this process unfolds, PP is reduced because of the dilution of nutrients in a wider mixing layer, and in accordance with our study, diversity increases as oligotrophic prokaryotes can appear in these nutrient‐poor waters (Álvarez‐Salgado et al. 2003). On the contrary, in summer–spring when nutrients arise from the depths to surface/shallow waters, the oligotrophs counterparts, the copiotrophs, which thrive in nutrient‐rich environments such as coastal upwelling zones (Pontiller 2021), are the prokaryotic species strongly proliferating in abundance during these events, causing lower prokaryotic richness and diversity. Moreover, the upwelling‐to‐downwelling transition characterised by wind relaxation events (Broullón et al. 2023) did not show significant differences in prokaryotic diversity compared to the precedent upwelling period. However, environmental conditions and physical forcing occurring from the beginning of upwelling towards the upwelling‐to‐downwelling transition only attenuate rather than drastically change abundance and diversity, promoting the proliferation of prokaryotes with different metabolic strategies, as it happens when copiotrophs start to decrease during the upwelling‐to‐downwelling transition period and turn into oligotrophic phylotypes when oceanographic conditions shift to downwelling. Foremost, the number of observed ASVs within each hydrographic period was more similar during upwelling pulses (234.05 ± 19.89) and upwelling‐to‐downwelling transition periods (250.14 ± 35.16), rather than those observed during downwelling (364.05 ± 76.67).

We also found significant differences in the distribution of the core‐prokaryotic assemblages at different taxonomic hierarchies (‘ASVs grouped’ within Class, Order and/or Family) driven by the changes induced by upwelling compared to downwelling periods. Indeed, our study identified three distinct microbial assemblages occurring in upwelling pulses, upwelling‐to‐downwelling transition and/or downwelling periods (Figure S7), in agreement with previous studies reporting a dynamic mosaic of prokaryotes responding to highly complex and dynamic hydrography associated with the upwelling (Allen et al. 2012; Bergen et al. 2015; Gregoracci et al. 2015; Zäncker et al. 2018; Bachmann et al. 2018). Some studies have found noticeably different communities during spring blooms (Lindh et al. 2017), while others have found the highest variability between winter and summer (Ward et al. 2017). In our study, the distinct community detected from May to September is likely a response to the upwelling pulses occurring in the area during this period (Figure 1). Indeed, temperature, salinity and organic material (PON and POC) (Figure S7) were identified as factors shaping upwelling and/or upwelling‐to‐downwelling transition communities. This is not surprising as temperature and prokaryotic abundance together with organic matter are the parameters that contribute the most to discerning microbial community shifts in this area during summer months (Montes et al. 2020). Moreover, there is strong evidence supporting the dominant role of temperature in prokaryotic community shaping (Mestre et al. 2020; Korlević et al. 2022), as temperature delimits nutrient restriction when the water column is stratified. Upwelling and upwelling‐to‐downwelling transition clusters shared a high relative abundance of ASVs affiliated with *Amylibacter*, *Planktomarina*, Cryomorphaceae, *Fluviicola*, Puniceispirillales and Flavobacteriales, which have been reported to be abundant in other upwelling areas (i.e., Orta‐Ponce et al. 2021; Costas‐Selas et al. 2023). Also, specific members of the other major prokaryotic group, Archaea MGII, increased during upwelling pulses, in accordance with previous results in the coastal waters of the NW Iberian upwelling, suggesting the fertilising effect of recently upwelled water on coastal bacterial assemblages (Montes et al. 2020). It is already well‐known that upwelling of North Atlantic Central Water, a water mass situated around 250‐500 m depth, occurs off the coast of Galicia during the summer season (already described in Fraga 1981). Previous studies have found that in this water mass the number of Archaea MGII can account for up to 10% of the total prokaryotic community in offshore waters between 250 and 500 m depth (Guerrero‐Feijóo et al. 2017) and likely arise in coastal waters during upwelling pulses. Furthermore, our study was able to detect a community shift from upwelling towards the upwelling‐to‐downwelling transition phase, with an associated increase in *Synechococcus* as the intensity of upwelling decreases, in agreement with other studies (Zhong et al. 2019), likely as a response to the changes in temperature and nutrient concentrations. Most importantly, we detect a significant increase in certain *Flavobacterium* genera, particularly *NS2b*, *NS4*, *NS5 and Formosa* during the upwelling‐to‐downwelling transition (i.e., ASV 235 belonging to NS2b or ASV 827 belonging to Formosa) compared to the previous upwelling period (ASV1792 belonging to NS2b or ASV200 belonging to Formosa), clearly suggesting that different marine Flavobacterial clades have distinct niches and different life strategies closely related to the environmental forcing (Figure 6B). The downwelling cluster was characterised by higher nutrient concentrations and precipitation (Table 1) and a considerable increase of ASVs belonging to Dadabacteriales, Marine Group II, Nitrosopumilaceae, Flavobacteria_NS9, *Prochlorococcus*, SAR11 or SAR406 was displayed. In this way, it is already well‐known that downwelling events extend the depth of the nutrient‐limited layer and the nutricline remains far below the phytoplanktonic reach, but highly dynamic circulation within a wide continental shelf can also induce the relocation of nutrient‐rich waters near the surface during downwelling hydrographic conditions (Han et al. 2021). Thus, our downwelling assemblages could be particularly sensitive to the variation of nutrient concentrations, as well as the addition of freshwater coastal runoff that may carry important organic material and nutrients offshore, and subsequently, the shifts in PP that may appear because of these processes (Espinoza‐González et al. 2012; Figueiras et al. 2020). Accordingly, our work displayed a peak in Dadabacteria from January to March–April in both 2017 and 2018 sampling years, significantly correlated with a huge precipitation peak detected in March 2018 and to a lesser extent in February–March 2017, in agreement with previous studies (Jokanović et al. 2021; Köhler et al. 2023). We further suggest that this seasonal variability found within the core‐phylotypes aforementioned is likely to be nothing more than the behaviour of a group of taxa at the ASV level. Thus, large intra‐diversity of closely related ASVs, adapted to different environmental conditions occupying temporally different niches is discussed further on in the next section.

### 4.4 Seasonal Communities Change Its ‘Closely Related ASVs‐Neighbors’ Under Upwelling‐Induced Disturbances

In addition to a first tentative description of a temporal succession of the dominant core‐prokaryotes, most importantly, our results allow us to identify temporal variability of closely related prokaryotic ASVs under upwelling‐induced disturbances to enable the assessment of the ecological relevance of the microdiversity (Figure 5). Overall, our data show that the highest microdiversity occurs among the most prevalent taxa compared to the rarely occurring taxa. For instance, this high microdiversity in the SAR11 clade or SAR116 clade (see Figure 6) should likely provide flexibility in terms of substrate utilisation (Larkin and Martiny 2017; García‐García et al. 2019) within those groups. Thus, we further hypothesised that this ecosystem maintained by some of these highly diverse taxa may have greater resilience to environmental/anthropogenic disturbances. Nevertheless, in our ecosystem also coexist other rare taxa with less microdiversity (i.e., *Fluviicola* or SAR86, see Figure 6). The latter could represent copiotroph organisms that are successful in short‐living in upwelling nutrient‐rich waters exploiting a narrow niche or a set of particular temporal upwelling conditions (UI, T, or PP, see Figure 7). Those variations in temporal distributions of closely related ASVs might highly inform us on ‘niche similarity’ among co‐occurring related taxa or ‘niche divergence’ among distinctively occurring ASVs. Precisely, closely related ASVs co‐varying could point to niche similarity, such as the 3 ASVs belonging Cryomorphaceae (ASV672, ASV900, ASV132) displaying high abundance in upwelling conditions and a significant positive correlation with T, UI and PON, thus, supporting their ability to exploit new nutrients (Tully et al. 2014; Montes et al. 2020). Specifically, the summer distribution of certain ASVs belonging to the previously mentioned *Fluviicola* (ASV1825, ASV207) is consistent with studies proliferating in locations relatively rich in organic carbon (Lee et al. 2019). Also, some niche similarity was found among the downwelling ASVs belonging to Dadabacteriales, showing negative correlations with T, total Chlorophyll, or particulate material (Graham and Tully 2020; Liu, Qiao, et al. 2022). Additionally, certain closely related ASVs belonging to Nitrosopumilaceae (ASV1464 and ASV648) and those belonging to SAR406 (ASV627 and ASV1884) become increasingly abundant in downwelling, likely reflecting niche overlapping as the correlation with environmental conditions is similar.

By contrast, other closely related ASVs present temporal differentiation, such as those belonging to Marine group II, showing completely different adaptations. For instance, ASV1183 occurrence (as an example of clear shift from previous ASV329 ecotype) might likely be associated with organic matter accumulation (Nieto‐Cid et al. 2006) and remineralisation processes, occurring after the bloom from late summer to winter (Bode et al. 2019). Furthermore, this ASVs differentiation within Marine group II may indicate different metabolic capabilities of particular ASVs of Archaea for transforming reworked organic material, as it has been previously reported (Wang et al. 2021). Other groups with closely related ASVs presenting temporal differentiation include SAR86 Clade or *Synechococcus*. In the case of SAR86 clade, there was a clear distinction between the downwelling HOC36 ASV becoming increasingly abundant between January and March, coherent with previous studies (Garate et al. 2022; Šantić et al. 2023) and SAR86 ASV becoming increasingly abundant in upwelling and upwelling‐to‐downwelling transition, likely as a result of the combination of top‐down processes and nutrient exhaustion (Dupont et al. 2011; Liu, Longnecker, et al. 2022). Moreover, different *Synechococcus* ASVs presented different adaptations. For instance, ASV1732 appeared all year round, while ASV853 and ASV1147 were abundant in upwelling‐to‐downwelling transition and ASV668 in the downwelling period. In this way, genomics of single cells have uncovered previously unknown marine microbial taxa and functions that have revealed the existence of new clades with distinct ecological and physiological adaptations and a high degree of genomic and functional diversity, likely allowing *Synechococcus* cells to optimally exploit a wide variety of spectral niches existing in marine ecosystems (Grebert et al. 2022).

### 4.5 Flavobacterium Genus: High Ecological Relevance in Upwelling Systems

Particularly interesting is the unveiling of the micro diversity hidden within the *Flavobacterium* genus, including several subtypes, compared to previous studies (Montes et al. 2020). Flavobacteria is an important class of Bacteroidetes, often making up a significant portion of marine microbial communities (Kirchman 2002), and it is not surprising that such a huge diversity is encountered in upwelling systems since they are found in both free‐living and attached to organic aggregates acting as major remineralisers of organic matter in these systems (Pontiller et al. 2022). Flavobacteriaceae microdiversity was very high in this study, with 24 ASVs occurring among the 6 most abundant genera (see Figure 6). This enormous microdiversity is not surprising, as previous studies concluded that Flavobacteriaceae are made of distinct extensive genomic variations (Tully et al. 2014; Gavriilidou et al. 2020; Kim et al. 2023). All these genomic changes could be associated with selective nutrient protein transporters (Davies et al. 2021) and/or cell surface biosynthesis (Morris et al. 2010) related to the success or failure of virus attachment along the temporal upwelling heterogeneity (Sowell et al. 2011). Overall, we found a clear distinction among seasonal ASVs (e.g., ASV993 belonging to NS9, ASV127 belonging to NS4 or ASV734 belonging to *NS5* marine group genus) and the ones appearing all year round (i.e., ASV1041_*Ulvibacter*, ASV1792 among others). Even those appearing all year round show seasonal variation in their relative abundance. Moreover, closely related ASVs belonging to the different *Flavobacterium* genera presented multiple variants with shared or distinctive environmental requirements. On one hand, *NS5*_ASV53, *NS2b*_ASV235 and ASV802 and NS9_ASV115 displayed a significant negative correlation with T, UI, PON, POC and TChl likely reflecting biological positive interactions and/or mirroring downwelling niche relatedness. On the other hand, *Formosa*_ASV827 and *NS4*_ASV671 were positively related to T, increasing in relative abundance with rising temperatures likely reflecting copiotroph oligotypes associated with coastal phytoplankton blooms further indicating potential temperature‐related changes to phytoplankton‐bacteria interactions (Arandia‐Gorostidi et al. 2020).

## 5 Conclusion

In this study, we report a clear pattern in microbial diversity, where specific core‐taxonomic groups shifted along with transitional hydrographic periods such as upwelling, upwelling‐to‐downwelling transition and downwelling. Furthermore, the implementation of fine‐scale taxonomical identification was crucial to explore and understand core‐taxa intra‐variability and their ecological implications. In this sense, our study has discovered seasonal differentiation for some closely related ASVs, before hidden at a broader taxonomic level (Valdés et al. 2021; Montes et al. 2020). Thus, for ecological studies, it is highly convenient to avoid merging taxa, because some of the observed variability of closely related ASVs differs in their patterns of correlations with the environmental requirements, and these differences might likely contribute to the function, biodiversity, and resilience of the marine microbiome. Overall, our results suggest the potential of environmental selection, particularly relevant in the face of climate change scenarios.

## Author Contributions

**Cessna‐Pamela Orta‐Ponce:** writing – original draft, methodology, formal analysis, data curation, visualization, investigation. **Rodrigo Alba‐Salgueiro:** formal analysis. **Carlota Rodríguez:** formal analysis. **Joaquín Valencia‐Vila:** formal analysis. **Pilar Díaz‐Tapia:** software. **Antonio Bode:** funding acquisition, writing – review and editing, project administration, resources. **Mar Nieto‐Cid:** supervision. **Marta M. Varela:** supervision, conceptualization, investigation, funding acquisition, writing – original draft, validation, visualization, writing – review and editing, project administration, resources.

## Conflicts of Interest

The authors declare no conflicts of interest.

## Supporting information


**Figure S1.** Location of station E2CO of A Coruña where all environmental variables were measured and water samples for the determination of the prokaryotic community composition and abundance were collected. Sampling took place once per month for a period of two years (May 2016–May 2018) as part of the RADIALES project.

**Figure S2.** Seasonality of environmental variables of subsurface water samples at station E2CO: (A) Temperature (°C), (B) Salinity, (C) particulate organic nitrogen, PON (mmol m‐3), (D) total chlorophyll, TChl (mg m3) and (E) nitrate, NO3 (mmol kg^−1^). Letters above the bars indicate the prevailing hydrographic periods: U = upwelling, T = transition, and D = downwelling.

**Figure S3.** Seasonal variability of accumulated precipitation from May 2016 to May 2018. Dots correspond with sampling dates. Fortnight averages of accumulated rain from MeteoGalicia metereological web historical data at Coruña‐Dique station. Letters above the bars indicate the prevailing hydrographic periods: U = upwelling, T = transition, and D = downwelling.

**Figure S4.** Average species diversity (H′) and estimated richness (S.Chao1) at upwelling, transition and downwelling events. Average values of each event were calculated by considering all months corresponding to the environmental event within the period of study. Error bars represent the standard error of Shannon diversity index (H′) and Chao richness estimator (S.Chao1).

**Figure S5.** Relative abundance (%) of core phylotypes from surface water samples (0 m) at station E2CO from May 2016 to May 2018. Colour shadow boxes and letters on the bottom indicate the upwelling (red), downwelling (blue) and transition (yellow) periods. ASVs grouped at different taxonomic levels appear in at least 50% of all samples and have a relative abundance higher or equal to 0.25.

**Figure S6.** Venn diagrams of prokaryotic groups between environmental events: upwelling, transition and downwelling. Prokaryotic groups were selected according the following criteria: one or the combination of more ASVs within each group appear in at least 50% of all samples and have a relative abundance higher or equal to 0.25. Samples of each environmental event were averaged for each ASV for each prokaryotic group. The Venn diagram illustrate the overlap abundance of ASVs found in these prokaryotic groups at upwelling, transition and downwelling events. The numbers in the circles indicate the number of unique ASVs in each event and the shared number of ASVs in the overlapping environmental events.

**Figure S7.** Redundancy analysis (RDA) biplot of prokaryotic diversity and environmental events—upwelling (U, green triangle), transition (T, blue inverted triangle) and downwelling (D, cyan square). Variables that showed multicollinearity are not shown. Each data point represents a prokaryotic community at a specific month. The direction of the arrows indicates the direction of maximum change of that variable, whereas the length of the arrow is proportional to the rate of change.

**Figure S8.** Heatmap show correlations among environmental and biological variables, and core‐phylotypes. The colour gradient indicates positive correlations in green tones and negative correlations in red tones, while black indicates no correlation. Significant correlations were highlighted with asterisks. Clusters show the aggregation of prokaryotic groups based on their similarity using Euclidian distance.

**Table S1.** Environmental characterisation of station E2CO over two years of study (May 2016–May 2018). Seawater temperature, salinity, density, nutrients (NO3, NO2, PO4 and SiO2), chlorophyll a, b, c1 + c2 and total concentrations (Chl‐a, Chl‐b, Chl‐c, TChl), primary production (PP), particulate organic carbon (POC), particulate organic nitrogen (PON), and particulate organic carbon and particulate organic nitrogen ratio (C:N ratio) were determined during the RADIALES project monthly sampling. Upwelling index (UI) and precipitation data was collected from the Rías Altas time‐series, and precipitation was obtained from the monthly accumulated rain data of Meteogalicia Coruña‐dique station.

**Table S2.** Results of marginal and sequential tests of distance based lineal model (DistLM) and the specified solution using the Akaike’s Information Criterion (AIC). *SS (trace)* is the sum of squares. *p* stands for *p*‐values. *res.df* are the degrees of freedom and *RSS* is the residual sum of squares. T: temperature; S: salinity, NO3 NO2: nitrate + nitrite; NO3 NO2: Phosphate; SiO2: silicate; TChl: total chlorophyll concentration; PP: primary production; POC: particulate organic carbon; UI: upwelling index.

## Data Availability

The data that support the findings of this study are available in Bioproject PRJNA1208201 at https://dataview.ncbi.nlm.nih.gov/object/PRJNA1208201, reference number PRJNA1208201. These data were derived from the following resources available in the public domain: https://dataview.ncbi.nlm.nih.gov/object/PRJNA1208201, https://dataview.ncbi.nlm.nih.gov/object/PRJNA1208201.
