# Supplementary material for: Microdiversity Shapes the Seasonal Niche of Prokaryotic Plankton Inhabiting Surface Waters in a Coastal Upwelling System
Source: Environ Microbiol Rep. 2025 Jul 21;17(4):e70131. doi: 10.1111/1758-2229.70131 (PMC12280048; doi:10.1111/1758-2229.70131)
Supplement: Supplementary file 1 — Figure S1. Location of station E2CO of A Coruña where all environmental variables were measured and water samples for the determination of the prokaryotic community composition and abundance were collected. Sampling took place once per month for a period of two years (May 2016–May 2018) as part of the RADIALES project. [file EMI4-17-e70131-s009.pdf]

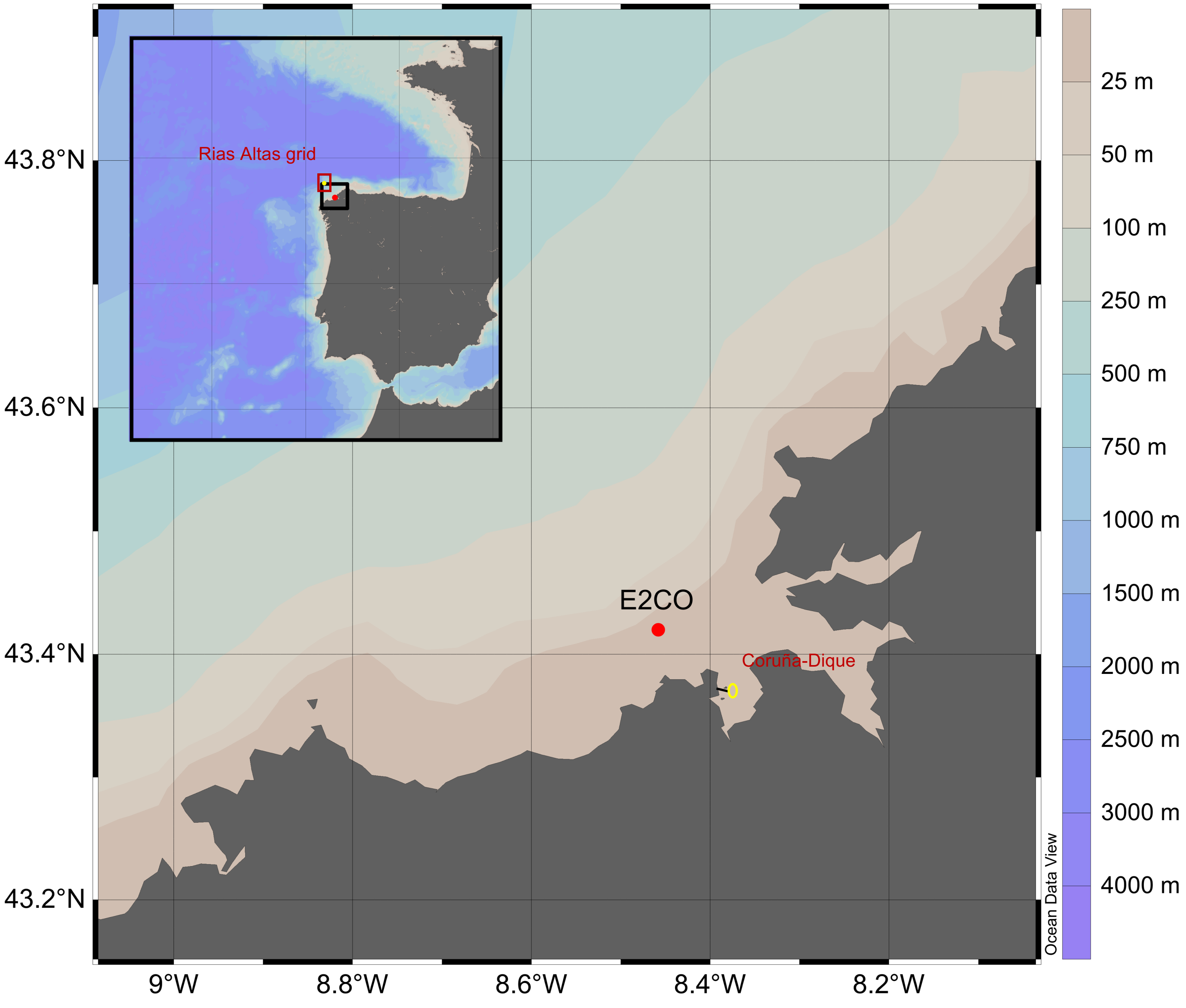

Figure S1. Location of station E2CO of A Coruña where all environmental variables were measured and water samples for the determination of the prokaryotic community were collected. Sampling took place once per month for a period of two years (May 2016-May 2018) as part of the RADIALES project.
