# Supplementary material for: Microdiversity Shapes the Seasonal Niche of Prokaryotic Plankton Inhabiting Surface Waters in a Coastal Upwelling System
Source: Environ Microbiol Rep. 2025 Jul 21;17(4):e70131. doi: 10.1111/1758-2229.70131 (PMC12280048; doi:10.1111/1758-2229.70131)
Supplement: Supplementary file 2 — Figure S2. Seasonality of environmental variables of subsurface water samples at station E2CO: (A) Temperature (°C), (B) Salinity, (C) particulate organic nitrogen, PON (mmol m‐3), (D) total chlorophyll, TChl (mg m3) and (E) nitrate, NO3 (mmol kg−1). Letters above the bars indicate the prevailing hydrographic periods: U = upwelling, T = transition, and D = downwelling. [file EMI4-17-e70131-s008.pdf]

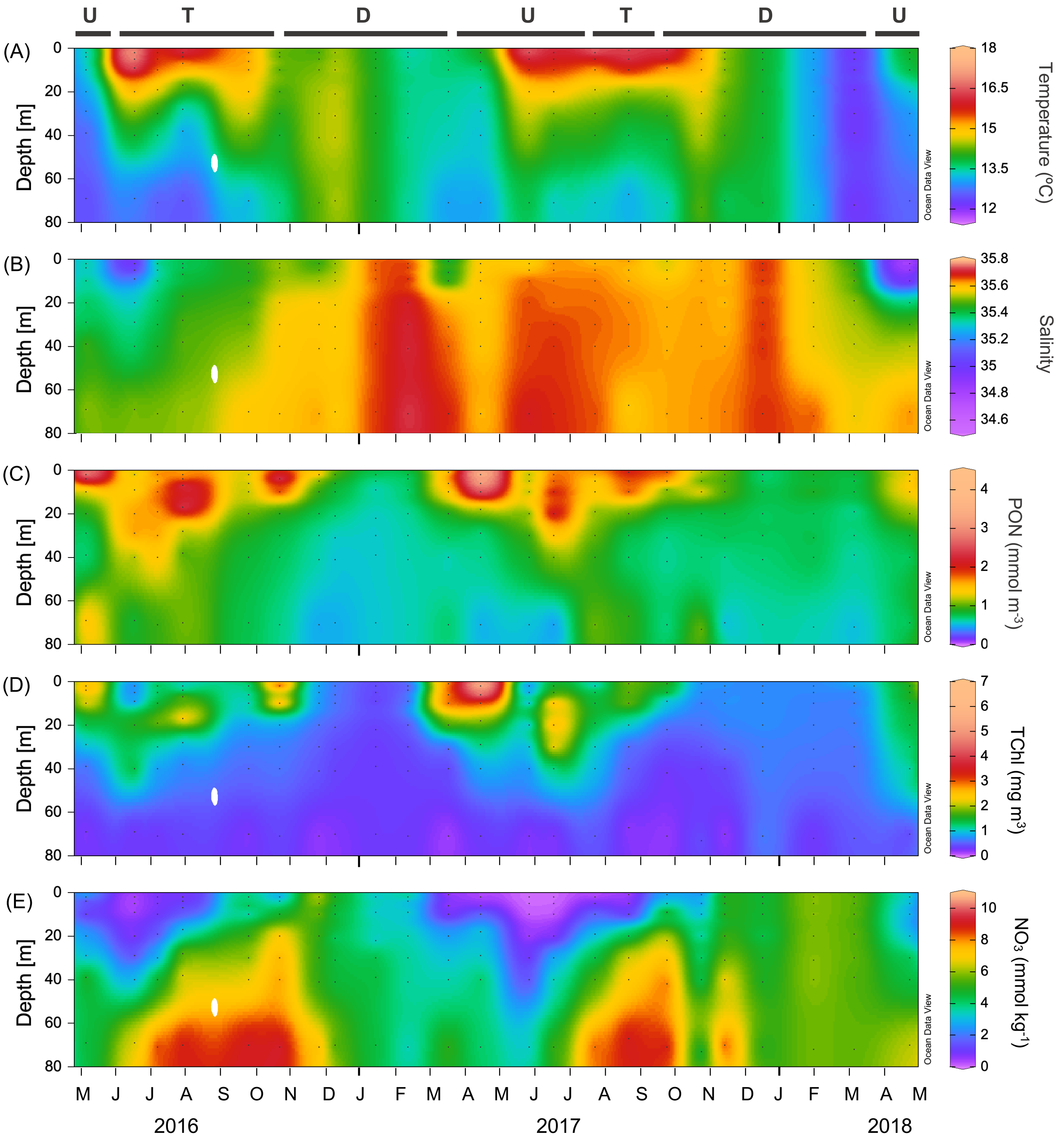

Figure S2. Seasonality of environmental variables of subsurface water samples at station E2CO: (A) Temperature (ºC), (B) Salinity, (C) particulate organic nitrogen, PON (mmol m^{-3}), (D) total chlorophyll, TChl (mg m^3) and (E) nitrate, NO_3 (mmol kg^{-1}). Letters above the bars indicate the prevailing hydrographic periods: U = upwelling, T = transition, and D = downwelling.
