# Supplementary material for: Microdiversity Shapes the Seasonal Niche of Prokaryotic Plankton Inhabiting Surface Waters in a Coastal Upwelling System
Source: Environ Microbiol Rep. 2025 Jul 21;17(4):e70131. doi: 10.1111/1758-2229.70131 (PMC12280048; doi:10.1111/1758-2229.70131)
Supplement: Supplementary file 3 — Figure S3. Seasonal variability of accumulated precipitation from May 2016 to May 2018. Dots correspond with sampling dates. Fortnight averages of accumulated rain from MeteoGalicia metereological web historical data at Coruña‐Dique station. Letters above the bars indicate the prevailing hydrographic periods: U = upwelling, T = transition, and D = downwelling. [file EMI4-17-e70131-s004.pdf]

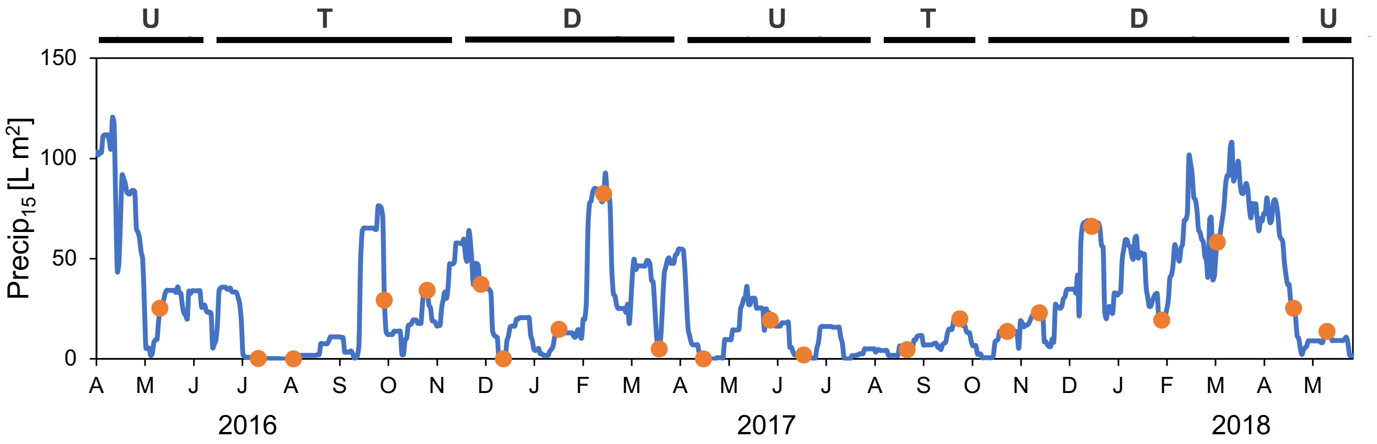

Figure S3. Seasonal variability of accumulated precipitation from May 2016 to May 2018. Dots correspond with sampling dates. Fortnight averages of accumulated rain from MeteoGalicia metereological web historical data at Coruña-Dique station. Letters above the bars indicate the prevailing hydrographic periods: U = upwelling, T = transition, and D = downwelling.
