# Supplementary material for: Microdiversity Shapes the Seasonal Niche of Prokaryotic Plankton Inhabiting Surface Waters in a Coastal Upwelling System
Source: Environ Microbiol Rep. 2025 Jul 21;17(4):e70131. doi: 10.1111/1758-2229.70131 (PMC12280048; doi:10.1111/1758-2229.70131)
Supplement: Supplementary file 4 — Figure S4. Average species diversity (H′) and estimated richness (S.Chao1) at upwelling, transition and downwelling events. Average values of each event were calculated by considering all months corresponding to the environmental event within the period of study. Error bars represent the standard error of Shannon diversity index (H′) and Chao richness estimator (S.Chao1). [file EMI4-17-e70131-s001.pdf]

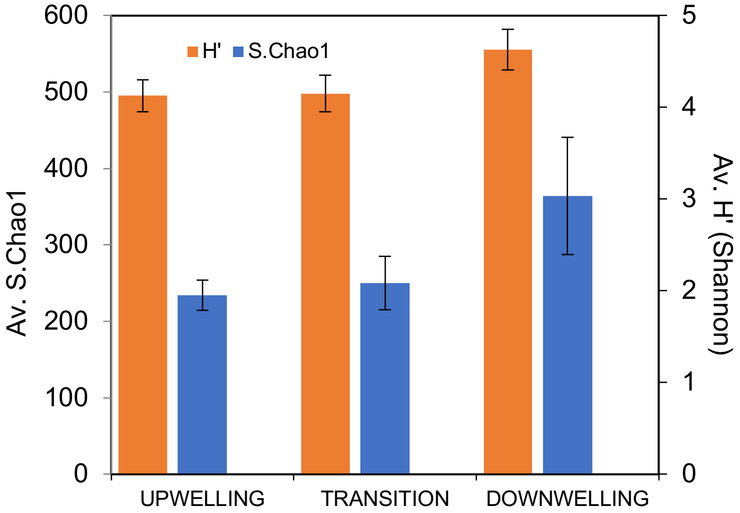

Figure S4. Average species diversity (H') and estimated richness (S.Chao1) at upwelling, transition and downwelling events. Average values of each event were calculated by considering all months corresponding to the environmental event within the period of study. Error bars represent the standard error of Shannon diversity index (H') and Chao richness estimator (S.Chao1).
