# Supplementary material for: Microdiversity Shapes the Seasonal Niche of Prokaryotic Plankton Inhabiting Surface Waters in a Coastal Upwelling System
Source: Environ Microbiol Rep. 2025 Jul 21;17(4):e70131. doi: 10.1111/1758-2229.70131 (PMC12280048; doi:10.1111/1758-2229.70131)
Supplement: Supplementary file 5 — Figure S5. Relative abundance (%) of core phylotypes from surface water samples (0 m) at station E2CO from May 2016 to May 2018. Colour shadow boxes and letters on the bottom indicate the upwelling (red), downwelling (blue) and transition (yellow) periods. ASVs grouped at different taxonomic levels appear in at least 50% of all samples and have a relative abundance higher or equal to 0.25. [file EMI4-17-e70131-s006.pdf]

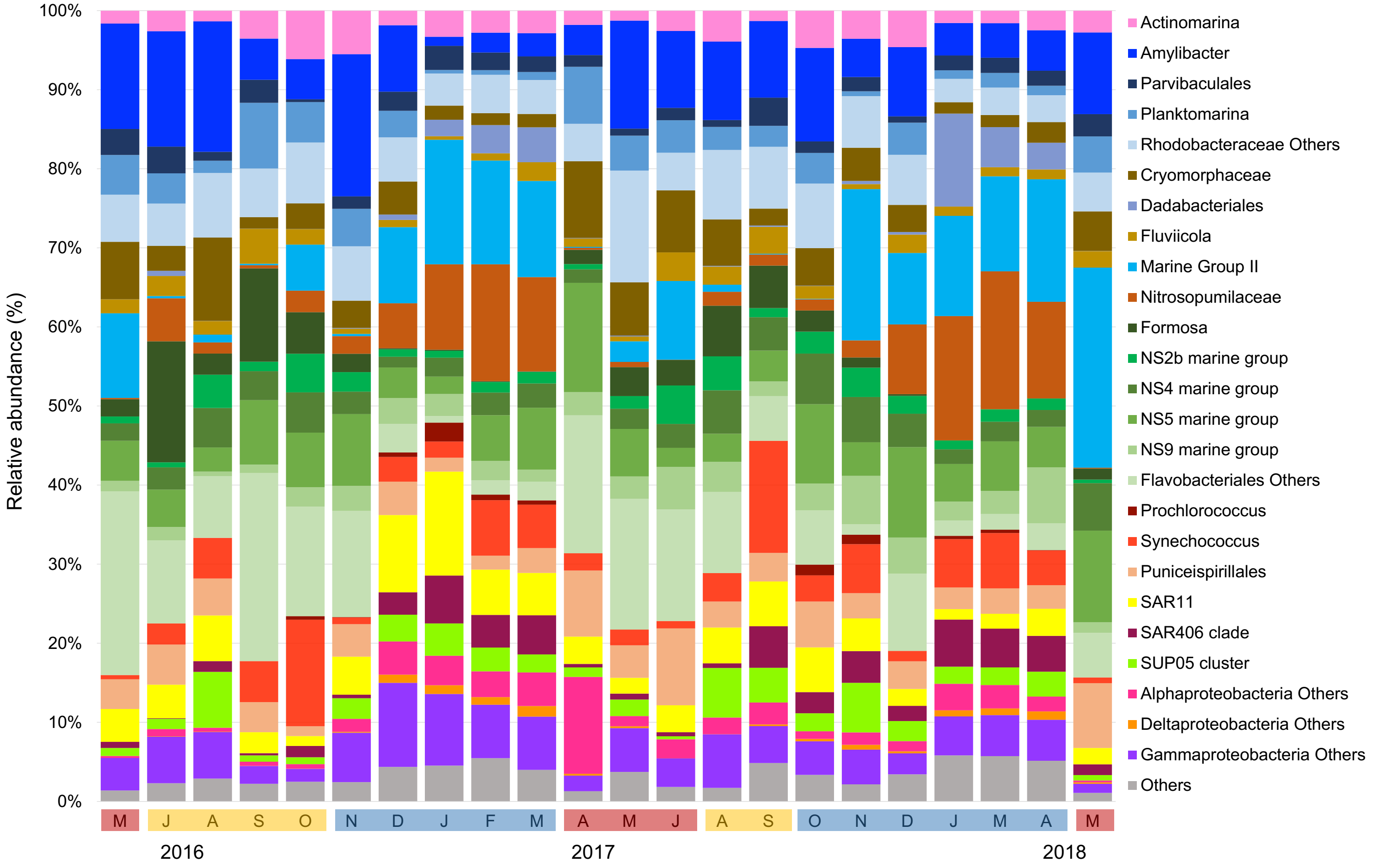

Figure S5. Relative abundance (%) of core phylotypes from surface water samples (0 m) at station E2CO from May 2016 to May 2018. Color shadow boxes and letters on the bottom indicate the upwelling (red), downwelling (blue) and transition (yellow) periods.
