# Supplementary material for: Microdiversity Shapes the Seasonal Niche of Prokaryotic Plankton Inhabiting Surface Waters in a Coastal Upwelling System
Source: Environ Microbiol Rep. 2025 Jul 21;17(4):e70131. doi: 10.1111/1758-2229.70131 (PMC12280048; doi:10.1111/1758-2229.70131)
Supplement: Supplementary file 6 — Figure S6. Venn diagrams of prokaryotic groups between environmental events: upwelling, transition and downwelling. Prokaryotic groups were selected according the following criteria: one or the combination of more ASVs within each group appear in at least 50% of all samples and have a relative abundance higher or equal to 0.25. Samples of each environmental event were averaged for each ASV for each prokaryotic group. The Venn diagram illustrate the overlap abundance of ASVs found in these prokaryotic groups at upwelling, transition and downwelling events. The numbers in the circles indicate the number of unique ASVs in each event and the shared number of ASVs in the overlapping environmental events. [file EMI4-17-e70131-s007.pdf]

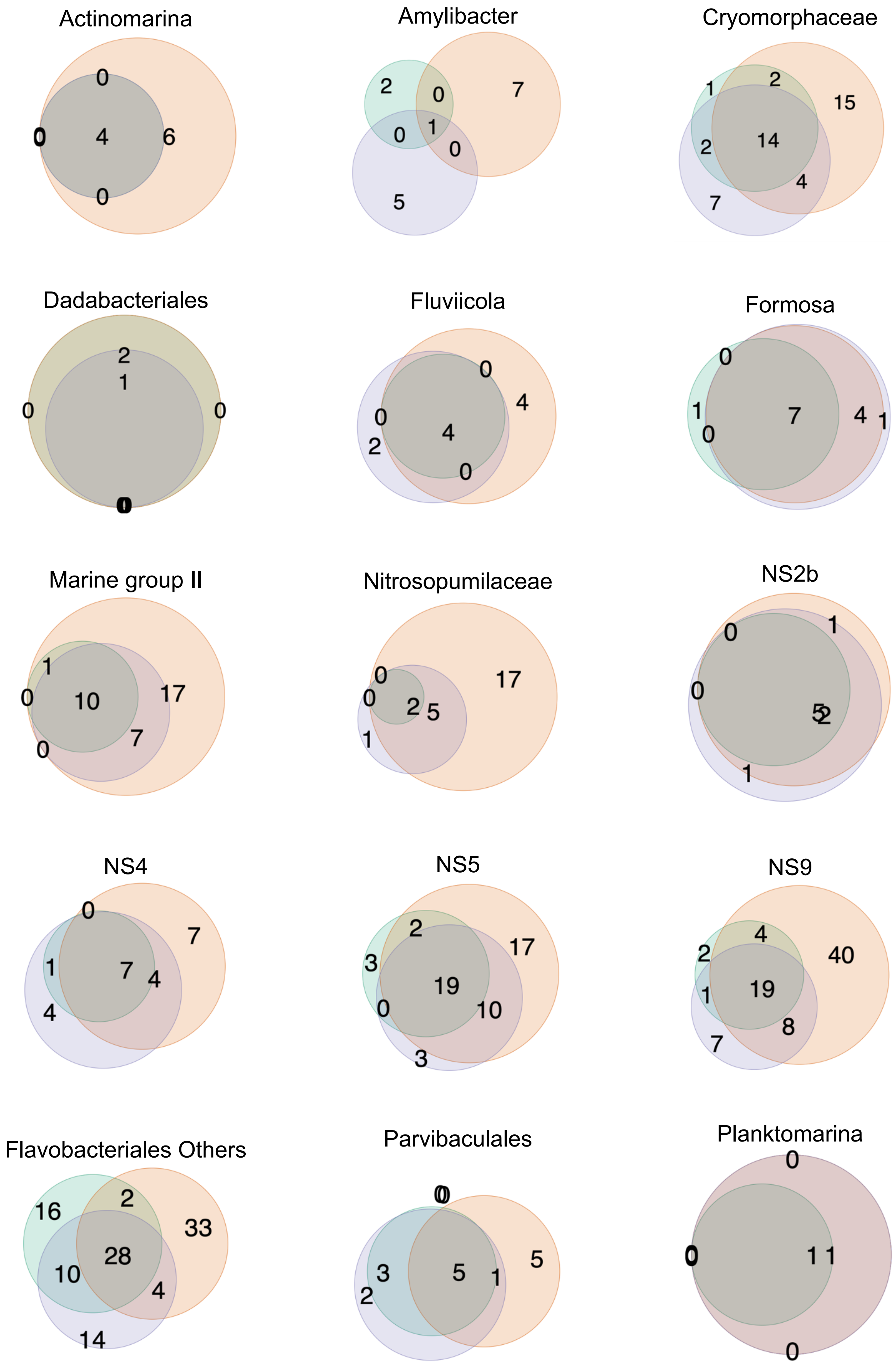
Actinomarina
0
0
4
6
0
Amylibacter
2
0
7
0
1
0
5
Cryomorphaceae
1
2
15
2
14
4
7
Dadabacteriales
2
1
0
0
Fluviicola
0
4
0
4
2
0
Formosa
0
1
7
4
1
0
Marine group II
1
0
10
17
7
0
Nitrosopumilaceae
0
17
0
2
5
1
NS2b
0
1
0
52
1
NS4
0
7
1
7
4
4
NS5
2
17
3
19
0
10
3
NS9
4
2
40
1
19
8
7
Flavobacteriales Others
16
2
33
28
10
4
14
Parvibaculales
0
3
5
1
5
2
Planktomarina
0
0
1
1
0

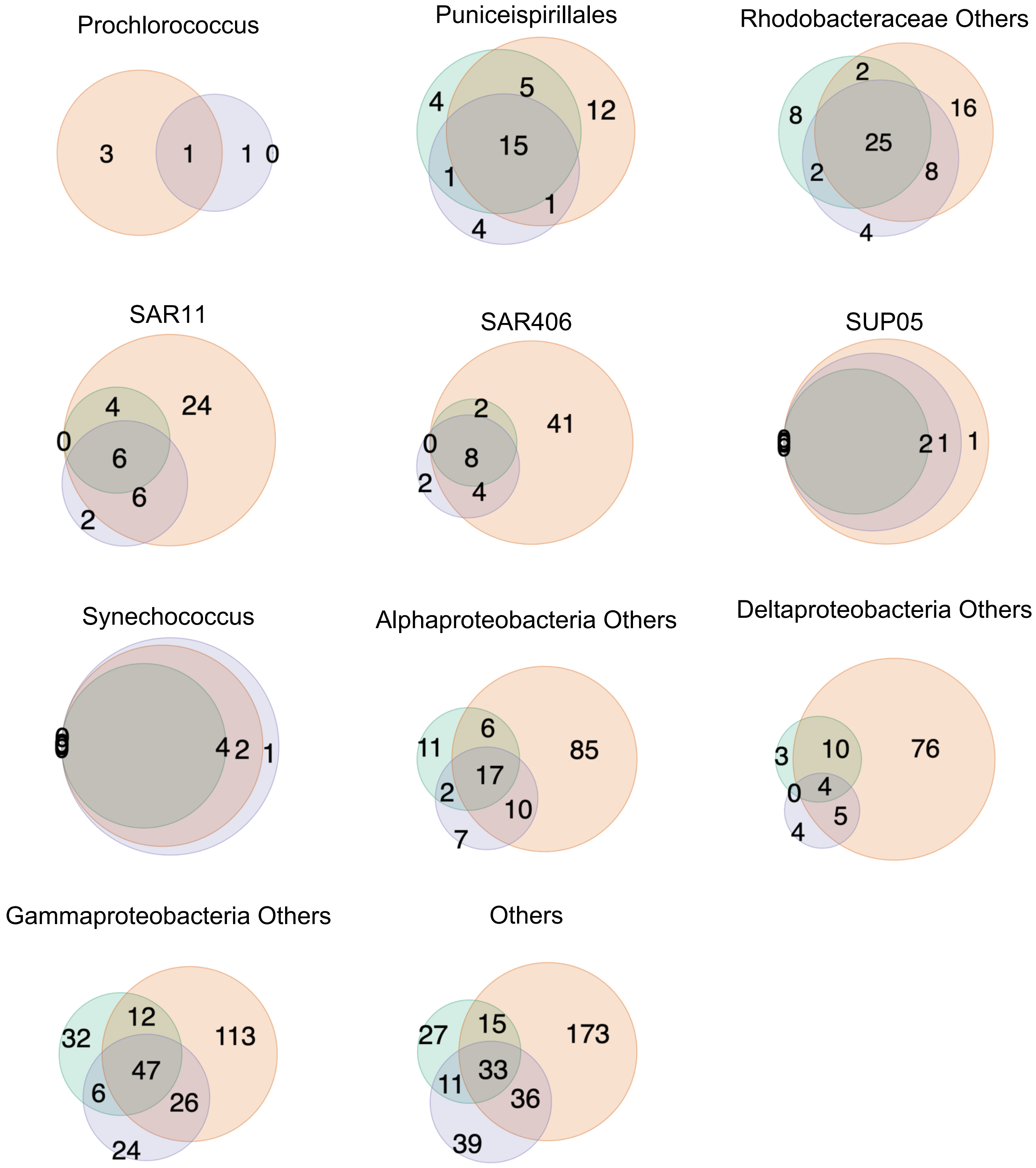

Figure S6. Venn diagrams of prokaryotic groups between environmental events: upwelling, transition and downwelling. Prokaryotic groups were selected according the following criteria: one or the combination of more ASVs within each group appear in at least 50% of all samples and have a relative abundance higher or equal to 0.25. Samples of each environmental event were averaged for each ASV for each prokaryotic group. The Venn diagram illustrate the overlap abundance of ASVs found in these prokaryotic groups at upwelling, transition and downwelling events. The numbers in the circles indicate the number of unique ASVs in each event and the shared number of ASVs in the overlapping environmental events.
