# Supplementary material for: Microdiversity Shapes the Seasonal Niche of Prokaryotic Plankton Inhabiting Surface Waters in a Coastal Upwelling System
Source: Environ Microbiol Rep. 2025 Jul 21;17(4):e70131. doi: 10.1111/1758-2229.70131 (PMC12280048; doi:10.1111/1758-2229.70131)
Supplement: Supplementary file 7 — Figure S7. Redundancy analysis (RDA) biplot of prokaryotic diversity and environmental events—upwelling (U, green triangle), transition (T, blue inverted triangle) and downwelling (D, cyan square). Variables that showed multicollinearity are not shown. Each data point represents a prokaryotic community at a specific month. The direction of the arrows indicates the direction of maximum change of that variable, whereas the length of the arrow is proportional to the rate of change. [file EMI4-17-e70131-s005.pdf]

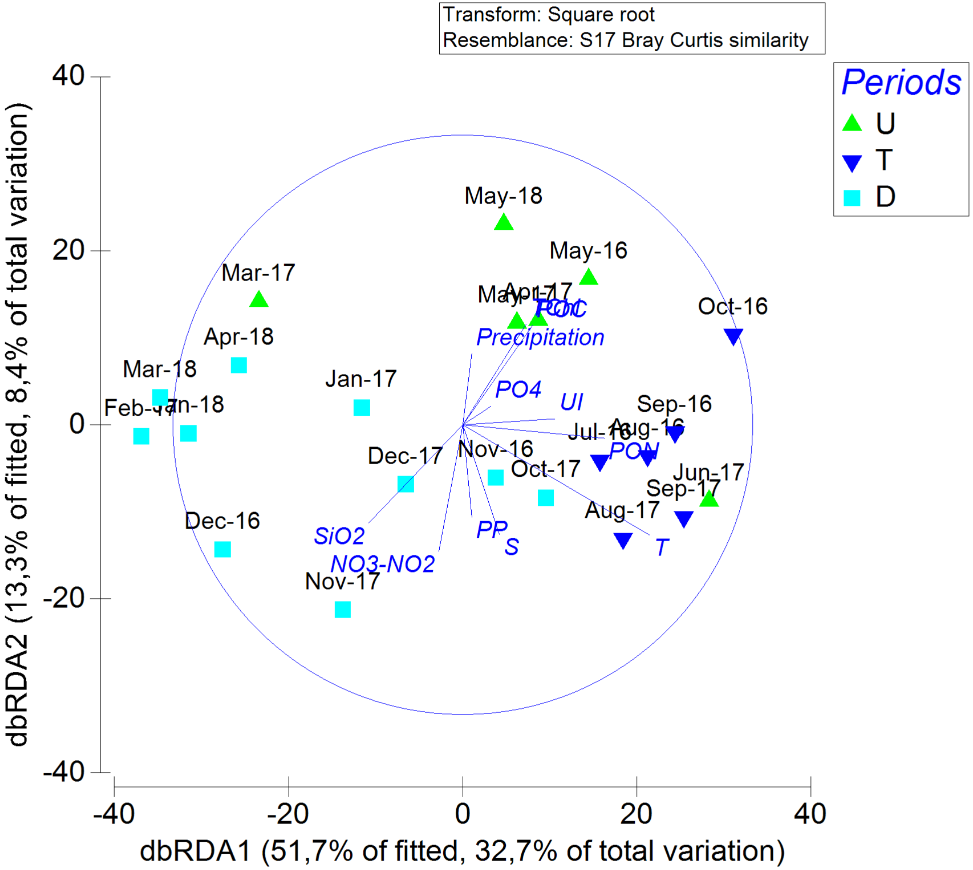

Figure S7. Redundancy analysis (RDA) biplot of prokaryotic diversity and environmental events - upwelling (U, green triangle), transition (T, blue inverted triangle) and downwelling (D, cyan square). Variables that showed multicollinearity are not shown. Each data point represents a prokaryotic community at a specific month. The direction of the arrows indicates the direction of maximum change of that variable, whereas the length of the arrow is proportional to the rate of change.
