# Supplementary material for: Microdiversity Shapes the Seasonal Niche of Prokaryotic Plankton Inhabiting Surface Waters in a Coastal Upwelling System
Source: Environ Microbiol Rep. 2025 Jul 21;17(4):e70131. doi: 10.1111/1758-2229.70131 (PMC12280048; doi:10.1111/1758-2229.70131)
Supplement: Supplementary file 8 — Figure S8. Heatmap show correlations among environmental and biological variables, and core‐phylotypes. The colour gradient indicates positive correlations in green tones and negative correlations in red tones, while black indicates no correlation. Significant correlations were highlighted with asterisks. Clusters show the aggregation of prokaryotic groups based on their similarity using Euclidian distance. [file EMI4-17-e70131-s003.pdf]

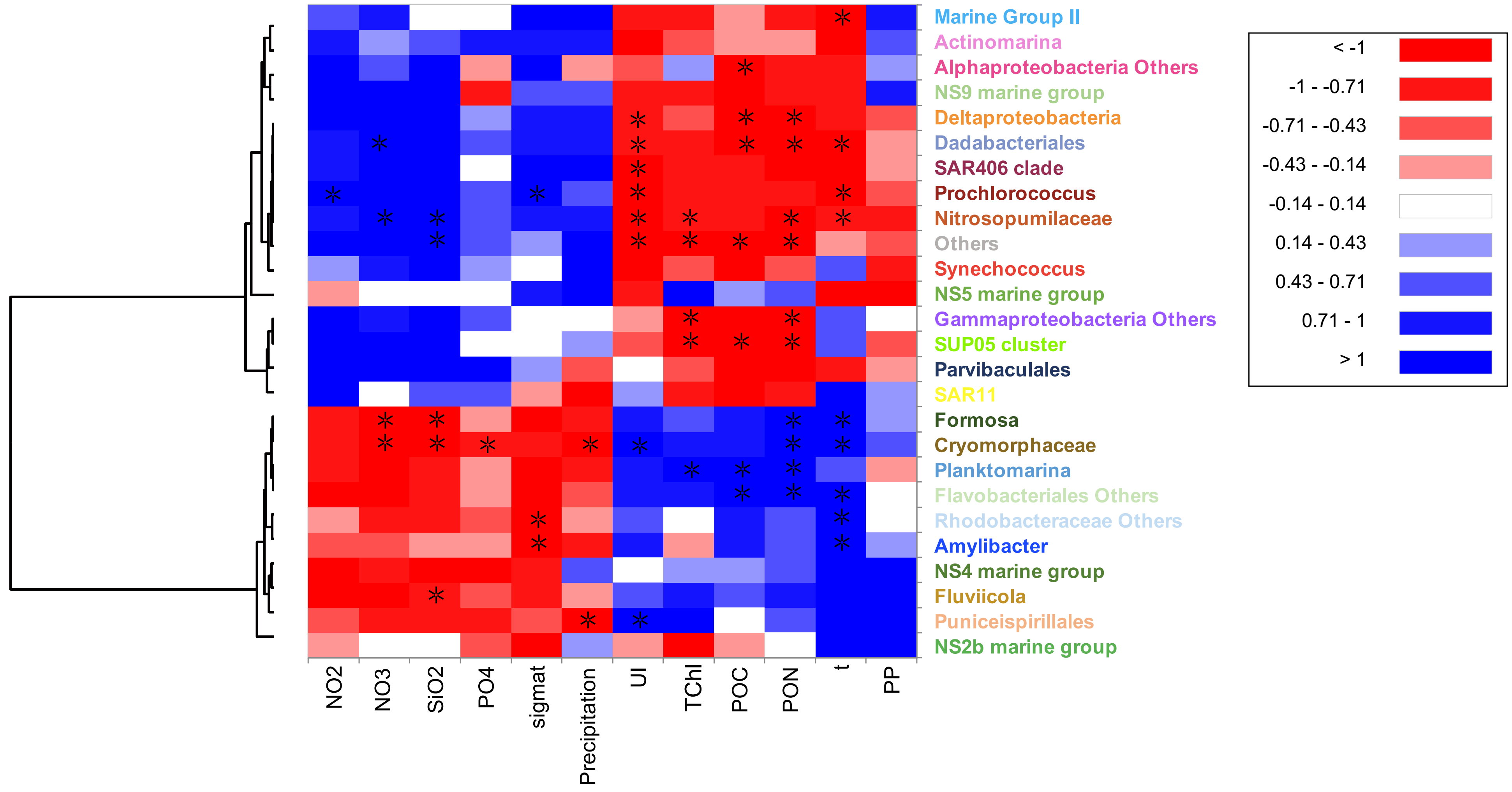

Figure S8. Heatmap show correlations among environmental and biological variables, and core-phylotypes. The color gradient indicates positive correlations in green tones and negative correlations in red tones, while black indicates no correlation. Significant correlations were highlighted with asterisks. Clusters show the aggregation of prokaryotic groups based on their similarity using Euclidian distance.
