# Supplementary material for: Microdiversity Shapes the Seasonal Niche of Prokaryotic Plankton Inhabiting Surface Waters in a Coastal Upwelling System
Source: Environ Microbiol Rep. 2025 Jul 21;17(4):e70131. doi: 10.1111/1758-2229.70131 (PMC12280048; doi:10.1111/1758-2229.70131)
Supplement: Supplementary file 9 — Table S1. Environmental characterisation of station E2CO over two years of study (May 2016–May 2018). Seawater temperature, salinity, density, nutrients (NO3, NO2, PO4 and SiO2), chlorophyll a, b, c1 + c2 and total concentrations (Chl‐a, Chl‐b, Chl‐c, TChl), primary production (PP), particulate organic carbon (POC), particulate organic nitrogen (PON), and particulate organic carbon and particulate organic nitrogen ratio (C:N ratio) were determined during the RADIALES project monthly sampling. Upwelling index (UI) and precipitation data was collected from the Rías Altas time‐series, and precipitation was obtained from the monthly accumulated rain data of Meteogalicia Coruña‐dique station. [file EMI4-17-e70131-s002.pdf]

Table S1. Environmental characterization of station E2CO over two years of study (May 2016-May 2018). Seawater temperature, salinity, density, nutrients (NO_3, NO_2, PO_4 and SiO_2), chlorophyll a, b, c1+c2 and total concentrations (Chl-a, Chl-b, Chl-c, TChl), primary production (PP), particulate organic carbon (POC), particulate organic nitrogen (PON), and particulate organic carbon and particulate organic nitrogen ratio (C:N ratio) were determined during the RADIALES project monthly sampling. Upwelling index (UI) and precipitation data was collected from the Rías Altas time-series, and precipitation was obtained from the monthly accumulated rain data of Meteogalicia Coruña-dique station.

Year	Month	Seawater temp. (ºC)	Salinity	Density (kg m^{-3})	NO_3 (mmol kg^{-1})	NO_2 (mmol kg^{-1})	PO_4 (mmol kg^{-1})	SiO_2 (mmol kg^{-1})	Chl-a (mg m^{-3})	Chl-b (mg m^{-3})	Chl-c (mg m^{-3})	TChl (mg m^{-3})	PP. (mgC m^{-3} h^{-1})	POC (mmol m^{-3})	PON (mmol m^{-3})	C:N ratio (molar)	UI (m^3 s^{-1} km^{-1})	Precipitation (L m^{-2})
2016	M	13.35	35.36	26.60	3.68	0.11	0.22	1.02	2.78	0.35	0.35	3.48	0.75	45.38	3.77	12.04	662.48	25.20
	J	15.24	35.49	26.29	0.98	0.06	0.17	0.61	1.56	0.07	0.26	1.89	8.74	14.07	1.81	7.77	596.35	0.20
	A	16.69	35.41	25.90	0.77	0.00	0.16	0.69	0.85	0.11	0.14	1.10	2.13	12.16	1.53	7.95	591.89	0.00
	S	15.47	35.44	26.21	4.09	0.25	0.38	2.91	0.95	0.25	0.10	1.30	1.97	7.56	1.10	6.87	-86.66	29.30
	O	14.15	35.53	26.57	1.61	0.10	0.28	1.31	3.81	0.20	0.61	4.62	5.03	40.54	4.50	9.01	30.17	34.20
	N	13.96	35.40	26.51	7.83	0.63	0.55	4.88	0.78	0.18	0.10	1.06	6.88	15.93	1.76	9.05	-222.37	37.20
	D	14.74	35.54	26.44	3.63	0.65	0.30	3.29	0.49	0.17	0.07	0.73	2.44	5.74	0.79	7.27	-372.53	0.00
2017	J	13.98	35.67	26.71	3.06	0.29	0.25	1.77	0.38	0.13	0.06	0.57	0.72	7.43	0.79	9.41	123.22	14.80
	F	13.34	35.68	26.85	4.64	0.45	0.33	1.85	0.31	0.10	0.06	0.47	2.39	5.81	0.76	7.64	-2487.11	82.40
	M	13.62	35.36	26.55	0.23	0.04	0.06	0.88	2.70	0.27	0.55	3.52	32.23	8.51	1.25	6.81	264.99	5.00
	A	13.87	35.62	26.69	0.20	0.04	0.08	0.87	6.84	0.39	1.18	8.40	0.68	21.81	4.19	5.21	1196.79	0.00
	M	17.12	35.53	25.89	0.00	0.04	0.04	0.39	0.20	0.05	0.04	0.29	1.29	10.65	0.88	12.10	181.49	19.40
	J	16.07	35.65	26.23	0.00	0.07	0.08	0.31	1.69	0.25	0.34	2.28	47.54	12.25	1.89	6.48	545.84	2.00
	A	16.45	35.64	26.14	0.10	0.08	0.00	0.97	1.97	0.40	0.28	2.65	34.03	11.76	2.21	5.32	504.34	4.60
	S	16.53	35.52	26.03	2.74	0.19	0.15	2.32	1.71	0.38	0.24	2.34	9.09	13.37	2.00	6.69	-290.86	20.00
	O	15.46	35.64	26.36	1.49	0.14	0.06	1.28	0.64	0.20	0.09	0.93	2.35	7.32	1.19	6.15	-704.75	13.80
	N	14.10	35.59	26.62	6.05	0.38	0.10	2.85	0.72	0.19	0.11	1.02	18.93	6.94	1.03	6.74	88.14	23.00
	D	13.89	35.69	26.74	4.50	0.29	0.14	2.18	0.70	0.27	0.11	1.08	8.09	4.80	0.64	7.50	-1139.28	66.20
2018	J	13.15	35.55	26.79	6.01	0.18	0.28	3.31	0.70	0.15	0.08	0.94	4.01	8.35	0.88	9.49	-914.83	19.40
	M	11.94	35.49	26.98	5.68	0.29	0.22	2.69	0.88	0.10	0.11	1.08	5.35	6.30	0.79	7.97	340.23	58.20
	A	13.93	34.53	25.84	4.71	0.24	0.15	3.23	0.84	0.14	0.11	1.09	5.22	6.10	0.87	7.01	-439.95	25.20
	M	13.96	35.25	26.39	0.03	0.04	0.10	0.44	3.58	0.19	0.71	4.48	21.14	16.42	2.14	7.67	210.51	13.80
