# Supplementary material for: Microdiversity Shapes the Seasonal Niche of Prokaryotic Plankton Inhabiting Surface Waters in a Coastal Upwelling System
Source: Environ Microbiol Rep. 2025 Jul 21;17(4):e70131. doi: 10.1111/1758-2229.70131 (PMC12280048; doi:10.1111/1758-2229.70131)
Supplement: Supplementary file 10 — Table S2. Results of marginal and sequential tests of distance based lineal model (DistLM) and the specified solution using the Akaike’s Information Criterion (AIC). SS (trace) is the sum of squares. p stands for p‐values. res.df are the degrees of freedom and RSS is the residual sum of squares. T: temperature; S: salinity, NO3 NO2: nitrate + nitrite; NO3 NO2: Phosphate; SiO2: silicate; TChl: total chlorophyll concentration; PP: primary production; POC: particulate organic carbon; UI: upwelling index. [file EMI4-17-e70131-s010.pdf]

Table S2. Results of marginal and sequential tests of distance based lineal model (DistLM) and the specified solution using the Akaike's Information Criterion (AIC). *SS (trace)* is the sum of squares. *P* stands for p-values. *res.df* are the degrees of freedom and *RSS* is the residual sum of squares. T: temperature; S: salinity, NO_3-NO_2: nitrate + nitrite; NO_3-NO_2: Phosphate; SiO_2: silicate; TChl: total chlorophyll concentration; PP: primary production; POC: particulate organic carbon; UI: upwelling index.

Marginal Tests:

Variable	SS (trace)	Pseudo-F	P	Prop.
**T**	5353.5	40.559	0.006	0.1686
**S**	1251.1	0.82036	0.654	3.94E+02
**NO_3-NO_2**	3470.3	24.541	0.029	0.10929
**PO_4**	1374.9	0.90521	0.442	4.33E-2
**SiO_2**	3568.4	25.323	0.034	0.11238
**TChl**	3027.4	21.078	0.055	9.53E+02
**PP**	1022.6	0.66556	0.714	3.22E+02
**POC**	4925	36.717	0.005	0.15511
**PON**	5134.9	38.583	0.006	0.16172
**UI**	2260.8	15.332	0.028	7.12E+02
**Precipitation**	1888.4	12.647	0.211	5.95E+02

Sequential Tests:

Variable	AICc	SS(trace)	Pseudo-F	P	Prop.	Cumul.	res.df
**T**	160.61	5353.5	40.559	0.001	0.1686	0.1686	20
**S**	162.49	971.96	0.7263	0.732	3.06E+02	0.19921	19
**NO_3-NO_2**	163.83	1863.5	14.235	0.175	5.87E+02	0.2579	18
**PO_4**	165.68	1606.3	12.436	0.229	5.06E+02	0.30849	17
**SiO_2**	168.25	1242	0.9593	0.418	3.91E+02	0.34761	16
**TChl**	168.33	3689.1	32.502	0.009	0.11618	0.46379	15
**PP**	172.03	1032.6	0.90392	0.547	3.25E+02	0.49631	14
**POC**	176.76	848.8	0.72862	0.725	2.67E+02	0.52304	13
**PON**	182.79	648.18	0.53657	0.884	2.04E+02	0.54346	12
**UI**	189.52	1060.2	0.86801	0.571	3.34E+02	0.57685	11
**Precipitation**	196.66	1782.6	15.297	0.144	5.61E+02	0.63299	10

Specified solution:

AIC	R^2	RSS	No.Vars	Selections
**196.66**	**0.6330**	11653	11	All

Percentage of variation explained by individual axes:

	% explained var. out of fitted model		% explained var. out of total variation	
**Axis**	**Individual**	**Cumulative**	**Individual**	**Cumulative**
**1**	51.68	51.68	32.71	32.71
**2**	13.26	64.94	8.39	41.1
**3**	7.68	72.62	4.86	45.97
**4**	6.51	79.13	4.12	50.09
**5**	4.77	83.89	3.02	53.1
**6**	4.42	88.31	2.8	55.9
**7**	3.11	91.42	1.97	57.87
**8**	2.85	94.27	1.8	59.67
**9**	2.47	96.74	1.57	61.24
**10**	2.16	98.9	1.37	62.61
**11**	1.1	100	0.69	63.3
